# Evaluation of a Curriculum‐Based Elementary School BikeSafe Program

**DOI:** 10.1111/josh.70162

**Published:** 2026-05-05

**Authors:** Michelina M. Witte, Alyson D. Brady, Hyuk Kim, TianHao Liu, Gillian A. Hotz

**Affiliations:** ^1^ The KiDZ Neuroscience Center at the Miami Project to Cure Paralysis, University of Miami Miller School of Medicine Miami Florida USA; ^2^ Department of Kinesiology and Sport Sciences University of Miami School of Education and Human Development Coral Gables Florida USA; ^3^ Department of Public Health Sciences Biostatistics Collaboration and Consulting Core University of Miami Miller School of Medicine Miami Florida USA

**Keywords:** bicycle safety, curriculum evaluation, elementary school, health equity, injury prevention, physical education

## Abstract

**Background:**

Children in urban areas face elevated risk for bicycle‐related injuries, yet few evidence‐based curricula address bicycle safety for elementary school physical education (P.E.) settings.

**Methods:**

The BikeSafe Elementary School Curriculum (BESC) is an off‐bicycle, standards‐aligned program for grades 2–5. From August 2024 to May 2025, P.E. teachers at nine Miami‐Dade County public elementary schools implemented the BESC with grades 2–3 (ages 7–8) and 4–5 (ages 9–11). Students completed pre‐post assessments. McNemar's test evaluated pre–post changes for each question; the Wilcoxon signed‐rank test assessed total score changes.

**Results:**

Data from 159 younger and 85 older students showed significant gains: grades 2–3 improved their knowledge assessment scores from 14.99 (SD = 2.18) to 16.70 (SD = 1.76, *p* < 0.001), and grades 4–5 from 21.80 (SD = 2.21) to 23.32 (SD = 2.01, *p* < 0.001). Both younger and older students improved on the concepts of visibility, hand signals, and built environment awareness.

**Implications for School Health Policy, Practice, and Equity:**

BESC's minimal equipment needs support integration into P.E. programs, particularly in underserved or high‐risk schools. Broader adoption may enhance youth injury prevention and safe cycling behaviors.

**Conclusions:**

BESC improved safety knowledge and is a scalable and alternative to on‐bike instruction in elementary school settings.

## 1 Introduction

Bicycling is a health‐enhancing activity for children, offering recreational enjoyment and a sustainable mode of transportation. It also contributes meaningfully to daily physical activity [1, 2], helping school‐aged children meet the U.S. Department of Health and Human Services' recommendation of at least 60 min of moderate‐to‐vigorous physical activity per day [3, 4]. Yet despite these benefits, bicycling in environments lacking safe infrastructure can pose serious injury risks [5]—particularly for younger, less experienced riders [6, 7].

Children are among the most vulnerable road users. Their smaller size, limited visibility, and developing cognitive and motor skills make them especially susceptible to traffic‐related injury. Globally, road traffic injuries (i.e., traffic crash injuries among all road users) claim the lives of an estimated 186,300 children each year, making it the fourth leading cause of death among children aged 5–9, third for those aged 10–14, and the leading cause of death among adolescents aged 15–17 [8]. Urban areas like Miami‐Dade County face heightened risks due to high vehicle traffic volumes and incomplete or unsafe bicycle infrastructure [9].

Despite this reality, formal bicycle safety instruction is rarely a standard component of elementary education in the United States [10], and education about how to safely navigate bike lanes and other transportation infrastructure is even less common, despite growing calls for infrastructure literacy in youth mobility education [11]. Many existing programs target older children [12, 13, 14], leaving a gap in developmentally appropriate instruction for elementary students. Moreover, programs that rely on bicycles, specialized equipment [15, 16] or dedicated facilities [17] face significant implementation barriers in under‐resourced schools. As a result, safety education is frequently omitted altogether rather than adapted, limiting both program reach and equity.

Emerging evidence supports the value of early, school‐based interventions, especially in the context of bicycle and pedestrian safety, injury prevention, or active transportation education [18]. Structured lessons can significantly improve children's knowledge of safe riding practices [13, 14, 19, 20, 21, 22, 23] including helmet use and traffic awareness, as well as their bicycling confidence [24] and self‐efficacy [25]. Importantly, these lessons do not need to rely on on‐bike activities. Off‐bicycle (“off‐bike”) instruction—delivered through interactive, movement‐based classroom or physical education (P.E.) activities—offers a scalable, comprehensive alternative that can be implemented across diverse school settings [13]. However, these programs are not yet common practice in U.S. elementary schools, which serve younger students. While at least 89 child‐focused bicycle safety education programs exist globally, their content, duration, and instructional strategies vary widely [26]. A 2019 comprehensive review found that few of these programs demonstrated measurable effectiveness, largely due to the absence of rigorous evaluation methods [26]. Additionally, a cross‐cultural study revealed that the age at which individuals learn to ride a bicycle varies by country and generation, with earlier learning observed in nations where bicycling is promoted through culture and infrastructure [27]. Taken together, these findings underscore the need for youth bicycle education programs to account for developmental stage, skill level, and age‐appropriate delivery and to vigorously evaluate their effectiveness.

The BikeSafe Elementary School Curriculum (BESC) was developed for pilot implementation in Miami‐Dade County public elementary schools, in partnership with local educators and transportation safety stakeholders. To support feasibility, sustainability, and broader applicability, the curriculum was intentionally aligned with state P.E. and health education standards. BESC uses a developmentally appropriate, off‐bike instructional approach to teach core bicycle safety concepts, including helmet fit, visibility, traffic rules, and features of safe street design such as painted versus protected bike lanes. Delivered through a “train‐the‐trainer” model, the curriculum can be implemented by P.E. teachers with minimal materials or equipment. Although initially piloted in Miami‐Dade County with support from the Florida Department of Transportation's Transportation Alternatives Program, the curriculum was designed to be scalable and adaptable for use in school districts beyond South Florida and in comparable urban settings.

This is the first study to evaluate a comprehensive, structured, off‐bike bicycle safety curriculum delivered as a P.E. program using a “train‐the‐trainer” model in U.S. elementary schools. The purpose of this study was to evaluate the effectiveness of the BESC in improving bicycle safety knowledge among students in grades 2–5. It was hypothesized that students would show statistically significant gains in bicycle safety knowledge, as measured by pre‐ and post‐intervention assessments.

## 2 Methods

### 2.1 Participants

#### 2.1.1 Demographics

School‐level demographic characteristics of participating sites are presented in Table S1 and reflect a cross‐section of the broader Miami‐Dade County Public Schools student population with respect to racial/ethnic composition, socioeconomic status, and sex distribution. All nine schools enrolled students from varied racial, ethnic, and socioeconomic backgrounds, providing a proxy for diversity and income‐related representation. Eligibility for free and reduced‐price lunch differed across participating schools (19%–80%), reflecting school‐level socioeconomic heterogeneity; the overall district‐wide prevalence in Miami‐Dade County Public Schools is approximately 73%.

#### 2.1.2 Recruitment Procedures

Schools were recruited through existing partnerships with the BikeSafe program and the University of Miami KiDZ Neuroscience Center, as well as through outreach conducted during professional development workshops orchestrated in collaboration with the school district's director of P.E. and health literacy and then via direct communication with school administrators and P.E. teachers. Eligible schools were selected based on expressed interest in piloting the curriculum, geographic diversity across all three Miami‐Dade County School Board regions, proximity to recent or planned bicycle and pedestrian infrastructure improvements, and elevated rates of bicycle–vehicle crash injuries in surrounding areas, as identified using Florida Department of Transportation crash data.

Student recruitment occurred at the classroom level within participating schools. All students in grades 2 through 5 (ages 7–11) enrolled in P.E. classes taught by participating teachers were eligible to participate. Information packets, including parental consent forms and child assent forms, were distributed to students through the schools prior to curriculum implementation. Participation was voluntary, and no incentives were provided. Students were included in the analytic sample only if they returned signed parental consent and child assent forms and completed both the pre‐ and post‐intervention assessments. A total of 1004 students were eligible and invited to participate. Because classes were delivered in combined grade bands (grades 2–3 and grades 4–5), eligibility was tracked at the grade‐band level rather than by individual grade. Of the eligible students, 442 (44.0%) returned signed parental consent and child assent forms, including 314 students in grades 2–3 and 128 students in grades 4–5.

Students were included in the analytic sample only if they returned signed consent and assent and completed both pre‐ and post‐intervention assessments. Ultimately, 159 students in grades 2–3 (50.6% of consented students) and 85 in grades 4–5 (66.4% of consented students) completed both the pre‐ and post‐test and were included in the final analysis. Although separate curricula were developed for grades 2–3 and 4–5, individual grade‐level effects were not examined because most P.E. class periods combined students within each grade band (2nd/3rd and 4th/5th grade), precluding disaggregated analysis.

### 2.2 Instrumentation

#### 2.2.1 Curriculum Development

The BESC was developed over 3 years using an iterative process informed by literature review, educator feedback, and alignment with Florida state standards. Separate curricula for grades 2–3 and 4–5 reflect developmental differences in cognitive skills and independence. It emphasizes helmet fit, visibility, traffic signs and signals, riding predictability, and interpretation of built environment features. Given that the built environment forms the foundation on which safe, slow, and complete streets exist [28, 29], the role of the built environment's design in the ultimate experience of safety by those riding their bikes was included. Teacher feedback informed revisions to improve clarity, usability, and feasibility within typical class periods, resulting in a field‐ready curriculum designed for diverse learners and school contexts. These modifications resulted in a field‐ready curriculum designed for diverse school contexts.

#### 2.2.2 Knowledge Assessments

Students completed a pre‐assessment prior to curriculum implementation and a post‐assessment upon curriculum completion. Question types included true/false, multiple choice, and matching items and were specific to grade level ranges. The 2nd–3rd grade version included 12 questions; the 4th–5th grade version included 20 questions. Each correct answer received one point. Students received de‐identified ID codes to enable matching of pre‐ and post‐assessments.

### 2.3 Procedure

#### 2.3.1 Early Feedback, Focus Groups, and Teacher Training

The curriculum was refined over a two‐year period through iterative pilot implementation, focus groups, and teacher feedback. Using a “train‐the‐trainer” model documented in both the WalkSafe elementary [30] and BikeSafe middle school [13, 31] programs, all participating teachers were given one‐on‐one training. Revisions focused on improving clarity, visual accessibility, and feasibility within standard class periods. Following refinement, the curriculum was implemented in participating schools for formal evaluation in year three.

#### 2.3.2 Curriculum Finalization, Description and Implementation

The key learning domains anchoring the final curriculum include: helmet fit, visibility, traffic signs and signals riding predictability, interpreting built environment features, and understanding what makes a safe environment for biking and walking. It includes three modules (*Before We Ride*, *Signs & Signals*, and *Safe Places*) each following a consistent instructional structure. Modules contain brief content overviews, key vocabulary, learning objectives, guided teacher scripts, and movement‐based activities supported by large visual aids designed for use in P.E. settings (see Supporting Information S2 for a representative sample of the grades 4–5 curriculum flipbook). Activities emphasize interactive, scenario‐based learning aligned with developmental level. Each module concludes with a review of key concepts and space for instructor reflection. This standardized structure supports feasibility, instructional consistency, and scalability across diverse school settings.

#### 2.3.3 Fidelity of Implementation

The team conducted site visits at participating schools using a standardized checklist. Observations confirmed adherence to the instructional format and module sequence. Curriculum completion forms were submitted by all teachers via the Qualtrics online platform.

### 2.4 Data Analysis

Prior to data collection, a power analysis allowed for the determination of the minimum sample size needed to be able to detect a 10% improvement in mean scores at 80% power and *α* = 0.05. Based on assumptions appropriate for paired, within‐subject analyses, a minimum of *n* = 42 paired observations per curriculum was required. The achieved analytic samples (grades 2–3: *n* = 159; grades 4–5: *n* = 85) exceeded this threshold.

The primary outcome was the change in total bicycle safety knowledge score within each grade band (grades 2–3 and grades 4–5), and confirmatory statistical inference was based exclusively on this endpoint. Because total scores represent paired, non‐normally distributed count data, pre–post differences were evaluated using the Wilcoxon signed‐rank test. Although results are summarized using means for ease of interpretation, the Wilcoxon test evaluates whether the distribution of paired differences differs significantly from zero.

Item‐level analyses were conducted as secondary, exploratory diagnostics to characterize content‐specific patterns of learning across curriculum domains. For individual binary items, McNemar's test was applied to assess paired changes in response accuracy. These analyses were not pre‐specified as confirmatory tests and are therefore reported descriptively using raw *p*‐values without adjustment for multiple comparisons. Item‐level findings are presented to contextualize patterns of learning rather than to support item‐wise inferential claims. All analyses were conducted using R (version 4.4.1).

## 3 Results

### 3.1 Overall Bicycle Safety Knowledge Gain

**FIGURE 1:**
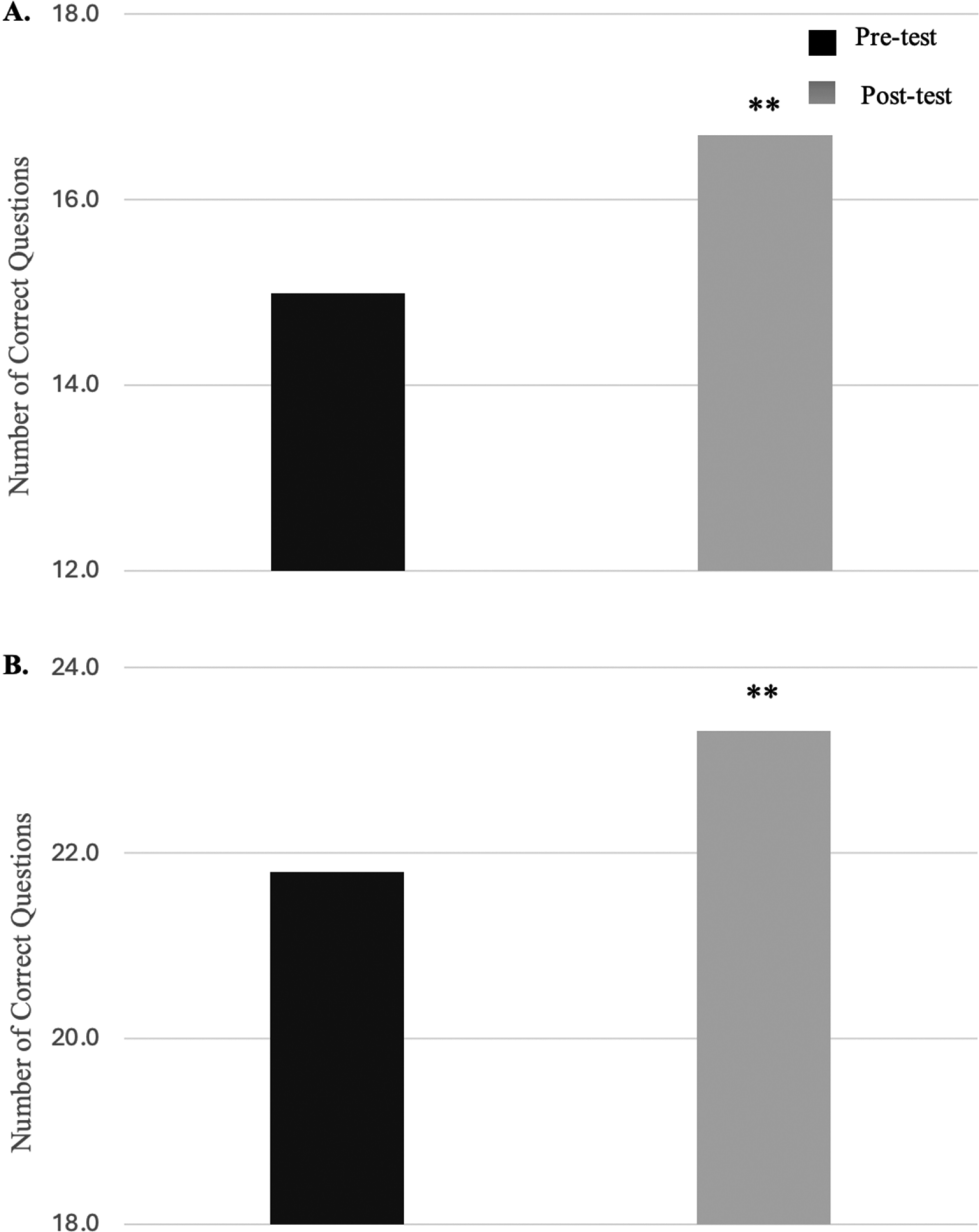
Overall knowledge score improvement by grade group. Pre‐ and post‐curriculum total mean scores on the BikeSafe knowledge assessment for students in grades 2–3 (Figure 1A, *N* = 159) and grades 4–5 (Figure 1B, *N* = 85). Scores increased significantly in both groups (**p < 0.001).

Following implementation of the BESC, students in both grade bands demonstrated significant improvements in their overall bicycle safety knowledge (Figure 1). In grades 2–3 (*N* = 159), the mean score increased from 14.99 (SD = 2.18) pre‐intervention to 16.70 (SD = 1.76) post‐intervention (*p* < 0.001, Figure 1A), reflecting a mean improvement of 1.71 points. In grades 4–5 (*N* = 85), the mean score increased from 21.80 (SD = 2.21) pre‐intervention to 23.32 (SD = 2.01) post‐intervention (*p* < 0.001, Figure 1B), reflecting a mean improvement of 1.52 points.

### 3.2 Item‐Level Improvements: Grades 2–3

**FIGURE 2:**
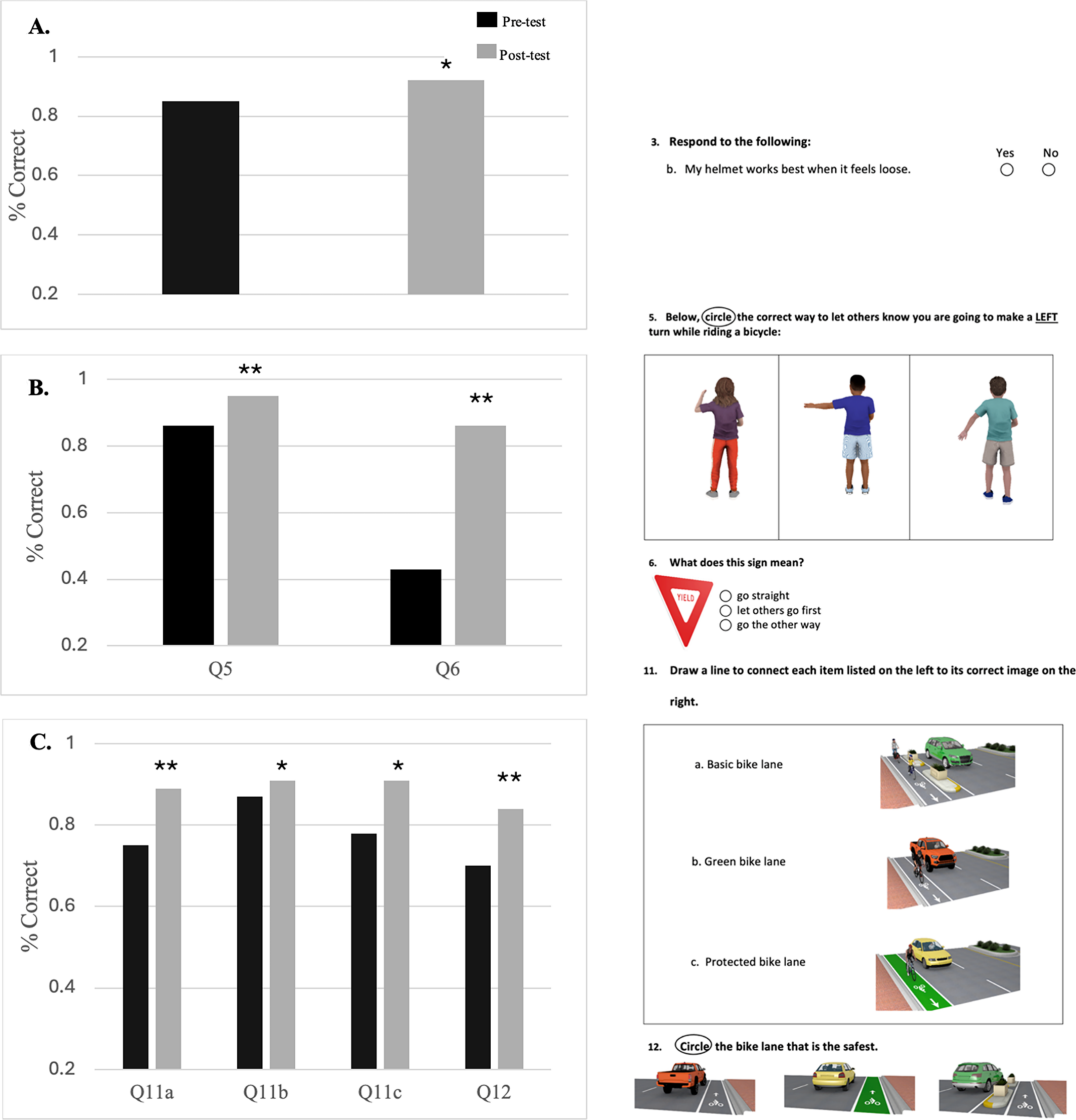
Question‐level knowledge gains: Grades 2–3. Mean change in scores on individual knowledge assessment items among children in grades 2–3. Outcomes are presented as item‐level proportions of correct responses. Key gains were observed on questions (visually shown at right) related to helmet fit (A, Q3b), signage and hand signals (B, Q5–Q6), and bike lane identification (C, Q11–Q12). Values represent item‐level proportions of students answering each question correctly; values are not components of a single total and therefore do not sum to 100%.

Significant item‐level knowledge gains were observed on the following test items: proper helmet fit (Q3b, Figure 2A), hand signals and road signs (Q5–Q6, Figure 2B), and safety concepts (Q9, vocabulary: “predictable,” Table 1). Improvements in young students' understanding of the built environment were shown in their knowledge improvements on questions relating to bike lanes and crosswalk safety (Q10a, Q10c, and Q10d, Table 1), bike lane types (Q11a–c, Figure 2C), and selecting the safest bike lane (Q12, Figure 2C). Notably, the questions related to helmet position (Q3a,c) and the purpose of hand signals (Q7) showed no improvement from pre‐ to post‐assessment (Table 1).

**TABLE 1:** BikeSafe Elementary Curriculum knowledge assessment broken down by question: Grades 2–3.

Q#	Question	Question type	Topic	Significant improvement	*p*
Q1	Do you know how to ride a bicycle or scooter?	Yes/no	Biking/scooting experience	N/A	N/A
Q2	What should you put on your head before you ride a bicycle? (*helmet*)	Multiple choice	Helmet safety	—	0.164
Q3a	I need to be sure my helmet properly fits my head before I ride. (*yes*)	Yes/no	Helmet fit	—	0.458
Q3b	My helmet works best when it feels loose. (*no*)	Yes/no	Helmet fit	**	0.012
Q3c	My helmet gets buckled under my chin. (*yes*)	Yes/no	Helmet fit	—	0.112
Q4	What can you wear to help others see you better when you ride a bicycle? (*a reflective vest*)	Multiple choice	Visibility	***	< 0.001
Q5	Below, circle the correct way to let others know you are going to make a LEFT turn while riding a bicycle (*three images shown of children making various hand signals*)	Multiple choice	Hand signals	***	< 0.001
Q6	What does this sign mean? [image of Yield Sign is shown] (*let others go first*)	Multiple choice	Signs and signals	***	< 0.001
Q7	*What is the safest way to cross the street?* (*look both ways before crossing and cross with a parent or trusted adult*)	Multiple Choice	Safe crossing	—	1
Q8	Using hand signals when riding my bicycle is important because it: (*helps others know what I'm about to do*)	Multiple choice	Predictability	*	0.015
Q9	Being *predictable* while riding my bicycle, means: (*others can easily guess my next move*)	Multiple choice	Predictability	***	< 0.001
Q10a	Fast cars make a street dangerous. (*yes*)	Yes/No	Safe environments	**	0.003
Q10b	Bike lanes are places where people can ride bikes. (*yes*)	Yes/No	Built environment	—	0.164
Q10c	Bike lanes are sometimes painted pink. (*no*)	Yes/No	Built environment	*	0.018
Q10d	Crosswalks are part of a safe street. (*yes*)	Yes/No	Safe environments	**	0.012
Q11a	Basic bike lane (*image of basic bike lane*)	Matching	Built environment	***	< 0.001
Q11b	Green bike lane (*image of green bike lane*)	Matching	Built environment	**	0.01
Q11c	Protected bike lane (*image of protected bike lane*)	Matching	Built environment	***	< 0.001
Q12	Circle the bike lane that is the safest [students choose from images of: unpainted bike lane, green painted bike lane, and protected bike lane]. (*protected bike lane*)	Multiple choice	Built environment	***	< 0.001
Total score	All questions	Mean scores from pre‐ to post‐curriculum		***	< 0.001

*Note:* This table displays the question number (Q#), question language, correct answers (in italics), the main topic being covered by the question and whether the mean scores for each bicycle safety assessment question among 2nd–3rd grade students significantly improved (*p < 0.05, **p < 0.01, or ***p < 0.001) after participating in the BikeSafe Elementary Curriculum.

### 3.3 Item‐Level Improvements: Grades 4–5

**FIGURE 3:**
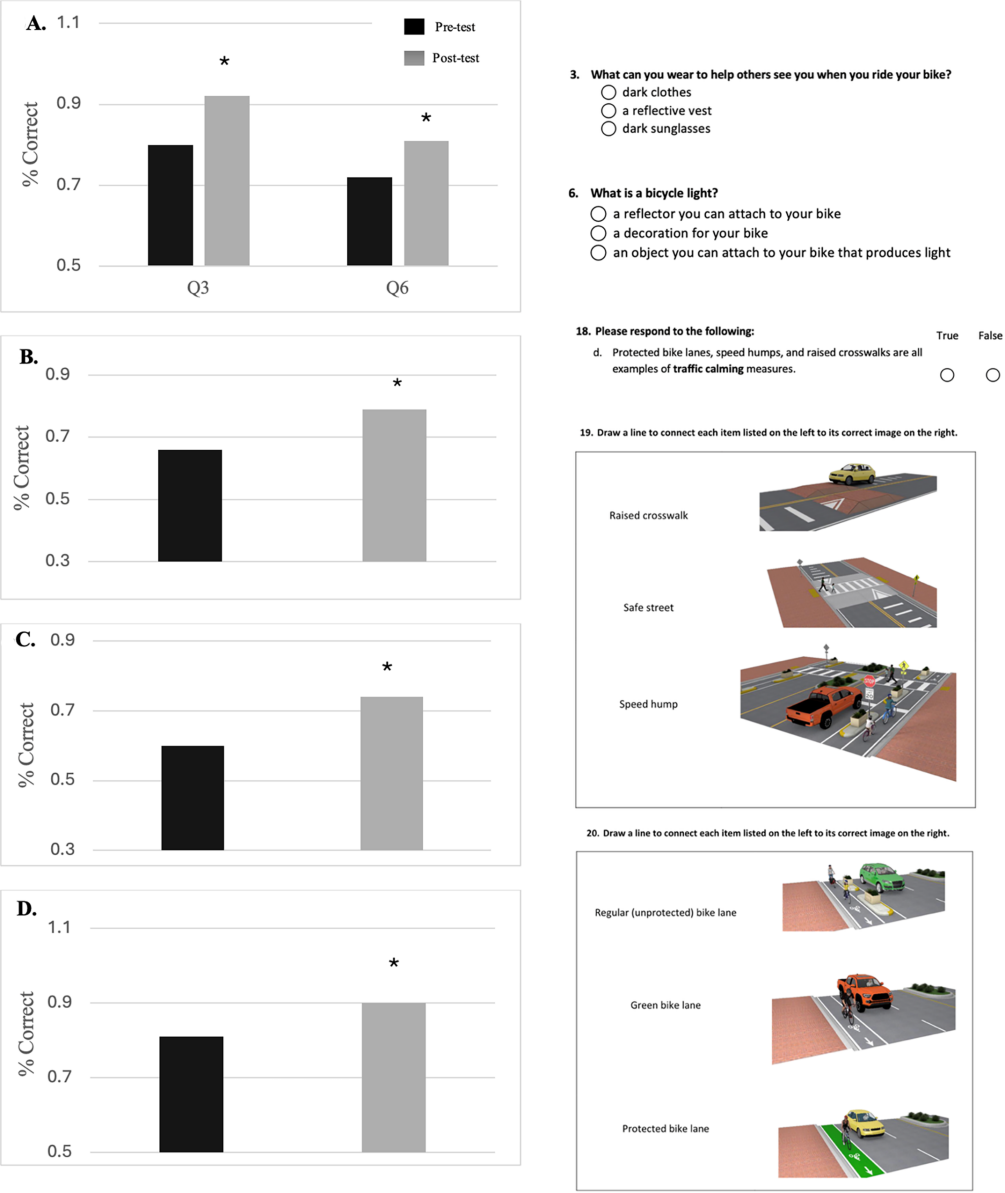
Question‐level knowledge gains: Grades 4–5. Mean change in scores on individual knowledge assessment items among children in grades 4–5. Outcomes are presented as item‐level proportions of correct responses. Key gains were observed on questions (visually shown at right) related to visibility strategies (A, Q3 and Q6), understanding of traffic calming measures (B,C, Q18d and Q19b), and identification of protected bike lanes (D, Q20c). Values represent item‐level proportions of students answering each question correctly; values are not components of a single total and therefore do not sum to 100%.

For older students, significant gains were modest but still meaningful. Specifically, significant item‐level knowledge gains were observed on the following test items: visibility and safety equipment by way of wearing bright clothing (Q3, Figure 3A) and the use of reflectors and bike lights (Q6, Figure 3A). These students also demonstrated improvements in their knowledge of hand signals by way of recognizing the correct signal (Q11, Table 2). Students improved their knowledge of the built environment and traffic calming as seen in by understanding calming measures (Q18d, Figure 3B), identifying safe street design (Q19b, Figure 3C), and the safest bike lane (Q20c, Figure 3D).

**TABLE 2:** BikeSafe Elementary Curriculum knowledge assessment broken down by question: Grades 4–5.

Q#	Question	Question type	Topic	Significant improvement	*p*
Q1	Do you know how to ride a bicycle?	Yes/no	Biking experience	N/A	N/A
Q2	To be safe when you ride your bike, you have to make sure: (*others can see you*)	Multiple choice	Visibility	—	1
Q3	What can you wear to help others see you better when you ride a bicycle? (*a reflective vest*)	Multiple choice	Visibility	**	0.004
Q4	What does it mean to be “visible?” (*being easily seen by others*)	Multiple choice	Visibility	—	0.061
Q5	What is a reflector? (*an object that bounces light back when light is shined on it*)	Multiple choice	Visibility	—	0.424
Q6	What is a bicycle light? (*an object you can attach to your bike that produces light*)	Multiple choice	Visibility	*	0.045
Q7	Bike lights are important when riding your bicycle because they: (*make you more visible and help you see where you're going*)	Multiple choice	Visibility	—	0.343
Q8	Wearing a helmet while riding your bike is important because (*it prevents head and brain injuries, if you were to fall*)	Multiple choice	Helmet safety	—	0.546
Q9	Below, circle the correct way to let others know you are going to make a LEFT turn while riding a bicycle (*three images shown of children making various hand signals*)	Multiple choice	Hand signals	—	0.149
Q10	Below, circle the correct way to let others know you are going to make a RIGHT turn while biking (*three images shown of children making various hand signals, with two correct answer choices, as two images show different ways to signal a right turn*)	Multiple choice	Hand signals	—	0.054
Q11	Below, circle the correct way to let others know you are STOPPING or SLOWING while biking (*three images shown of children making various hand signals*)	Multiple choice	Hand signals	***	< 0.001
Q12	What is the safest way to cross the street when riding your bike? (*look both ways and cross when it is your turn*)	Multiple choice	Safe intersection navigation	—	1
Q13	Why should you use hand signals and follow traffic rules when riding your bike? (*because it helps others around me know what I am about to do*)	Multiple choice	Predictability	—	0.606
Q14	What does it mean to be *predictable* while riding your bike? (*to ride in a way that others expect you to ride*)	Multiple choice	Predictability	—	0.689
Q15	Why is it dangerous when drivers make a right turn when the traffic light is red? (*because drivers turning right on red do not see people walking or biking at intersections*)	Multiple choice	Safe intersection navigation	—	0.067
Q16	What is a protected bike lane? (*a bike lane separated from traffic by a physical barrier*)	Multiple choice	Built environment	—	0.361
Q17	Traffic calming measures work to: (*slow down cars in areas where people ride bikes, walk or roll*)	Multiple choice	Built environment	—	0.423
Q18a	A safe street is designed so people can walk, roll, or ride bikes on it without getting hurt. (*true*)	True/false	Safe environment	—	0.099
Q18b	Protected bike lanes are safer than regular bike lanes because they separate bicycle riders from car drivers. (*true*)	True/False	Built Environment	—	1
Q18c	Traffic calming measures encourage drivers to speed up where people are riding, walking, or rolling. (*false*)	True/false	Built environment	—	0.383
Q18d	Protected bike lanes, speed humps, and raised crosswalks are all examples of traffic calming measures. (*true*)	True/false	Built environment	*	0.037
Q19a	Draw a line to connect each item listed on the left to its correct image on the right. (*choose from images of: raised crosswalk, safe street, and speed hump*) Raised crosswalk	Matching	Built environment	—	0.052
Q19b	Safe street	Matching	Built environment	**	0.004
Q19c	Speed hump	Matching	Built environment	—	0.302
Q20a	Draw a line to connect each item listed on the left to its correct image on the right. (*choose from images of: unpainted bike lane, green painted bike lane, and protected bike lane*) Regular (unprotected) bike lane	Matching	Built environment	—	0.066
Q20b	Green bike lane	Matching	Built environment	—	0.077
Q20c	Protected bike lane	Matching	Built environment	*	0.01
Total score	All questions	Mean scores from pre‐ to post‐curriculum		***	< 0.001

*Note:* This table displays the question number (Q#), question language, correct answers (in italics), the main topic being covered by the question and whether the mean scores for each bicycle safety assessment question among 4th–5th grade students significantly improved (*p < 0.05, **p < 0.01, or ***p < 0.001) after participating in the BikeSafe Elementary Curriculum.

### 3.4 Overall

Younger students (grades 2–3, Table 1) demonstrated broader improvements across more items than older students (grades 4–5, Table 2), likely reflecting lower baseline knowledge. Both groups improved on key domains including visibility, hand signals, and built environment awareness, aligning with the curriculum's intended learning objectives.

## 4 Discussion

This study demonstrates that overall bicycle safety knowledge improved among students in grades 2–5 following implementation of the BESC, with the most consistent and robust gains observed among younger students (grades 2–3), particularly in core safety domains such as helmet fit, visibility, hand signals, traffic signs and signals, and recognition of safe and supportive built environment features. These results underscore the value of off‐bike instruction in improving safety outcomes.

While students across all grade levels demonstrated significant overall gains in bicycle safety knowledge, gains were most pronounced among younger students, which may reflect greater developmental receptivity to structured safety instruction, lower baseline familiarity with traffic concepts, and increased opportunity for measurable learning gains at earlier stages of cognitive and motor development. In contrast, older students (grades 4–5) entered the intervention with higher baseline knowledge for some safety domains, which may have limited observable gains on certain assessment items. Specifically, three items in the grade 4–5 assessment (Q2, Q10, and Q13, Table 2) did not show significant pre‐to‐post improvement. This may reflect a ceiling effect, as baseline knowledge for certain safety concepts—such as visibility and hand signals—was already high among older students, thereby limiting the measurable range for gains. Ceiling effects are common in educational and health‐related interventions when participants enter with baseline knowledge, which can reduce measurable item‐level change, despite meaningful reinforcement of learning [32]. Future evaluations may benefit from incorporating more challenging assessment items or expanded response options for older students to better capture incremental knowledge gains.

The off‐bike format of the BESC is a major strength. It eliminates the need for bicycles or specialized infrastructure, making it easily implementable. Its low material burden allows for broad dissemination—especially in schools located in urban areas with high rates of child cyclist injuries. In Title I schools, which often face systemic underinvestment in infrastructure and health education [33], scalable, standards‐aligned safety programs may help mitigate disproportionate injury risks. Furthermore, by focusing on interactive, movement‐based activities that do not require bicycles, the curriculum ensures equitable access to safety education.

The program aligns with broader initiatives such as Vision Zero [34] and Safe Routes to School [6] by reinforcing traffic awareness, infrastructure literacy, and safe riding behaviors—skills that empower students as road users and future multimodal travelers. The curriculum can also be used in tandem with built environment improvements which have been shown to encourage community support for safe streets [35], such as pop‐up bike lanes or traffic‐calming demonstrations. As noted by Cushing et al. [34], such multi‐layered approaches support a comprehensive and coordinated framework for advancing road safety and public health outcomes. Particularly in areas with limited access to safe transportation infrastructure, they serve as a critical component of multi‐sector injury prevention [36].

As communities promote active transportation—including e‐bikes, scooters, and other emerging modes—scalable, equitable, evidence‐based tools like BikeSafe are essential to ensure safety keeps pace with mobility access. The BESC helps ensure children develop foundational safety knowledge early, helping them stay not only active but safe as they transition to increasingly complex and high‐speed mobility options. Widespread adoption of off‐bike safety curricula, especially in coordination with built environment improvements and transportation equity policies, represents a critical step toward safer mobility for all.

In summary, the BESC significantly improved bicycle safety knowledge especially among younger students—while addressing barriers that limit access to traditional, on‐bike programs. Its low‐resource, developmentally tailored, and culturally responsive design makes it well‐suited for scalable integration into P.E. curricula, including in under‐resourced schools.

### 4.1 Implications for School Health Policy, Practice, and Equity

Findings support the inclusion of structured bicycle safety education into elementary school P.E. and health curricula. Early, developmentally appropriate instruction may improve foundational safety knowledge before independent riding behaviors are established.

From a practice perspective, the off‐bike design addresses key implementation barriers by eliminating the need for bicycles, specialized equipment, or dedicated facilities. This increases feasibility in schools with limited resources and supports delivery within standard P.E. classes.

From an equity standpoint, the curriculum's minimal resource requirements make it accessible to schools serving populations where infrastructure and programmatic resources may be limited. By embedding safety education within existing school structures, the BESC provides a scalable approach to reducing disparities in access to injury prevention education.

### 4.2 Limitations

Several limitations should be considered when interpreting the findings. First, the study employed a single‐group pre–post design without a comparison group, which limits causal inference and precludes ruling out potential confounding factors such as maturation or external influences. This design was selected to evaluate curriculum feasibility and effectiveness under real‐world school conditions, where randomization or withholding safety education from eligible students was not feasible nor ethically appropriate. Future evaluations should incorporate comparison or control groups, such as matched schools, staggered implementation designs, or cluster‐randomized approaches, to strengthen causal inference and better isolate curriculum effects from external influences.

Second, the evaluation relied on pre‐ and post‐intervention knowledge assessments and did not measure behavioral outcomes, injury rates, or long‐term knowledge retention. While improvements in safety knowledge are a critical precursor to safer behavior, future research should examine whether these gains translate into sustained behavior change and reduced injury risk over time. Evaluations assessing behavioral skills, such as hazard recognition, decision‐making in simulated environments, or observed riding behaviors, would provide important insight into whether knowledge gains from the BESC translate into safer real‐world practices. Longitudinal follow‐up studies assessing knowledge retention and skill progression are warranted.

Third, item‐level analyses were exploratory and designed to provide insight into content‐specific patterns of learning rather than item‐wise inferential claims. In the older students, some assessment items did not demonstrate statistically significant improvement, which may reflect ceiling effects due to high baseline knowledge. More sensitive or challenging assessment tools may be needed to capture incremental gains among older students.

Fourth, the study was conducted within a single large urban school district, which may limit generalizability to rural or suburban contexts with different transportation environments and educational structures. Future studies conducted across varied geographic and demographic settings would strengthen external validity and inform broader dissemination.

Logistical constraints common to school‐based research—such as absences, scheduling variability, and incomplete assessments—reduced the analytic sample size relative to initial recruitment. For these reasons, participant attrition and missing data represent an important limitation. Although 1004 students were initially recruited across participating schools, only students with complete pre‐ and post‐intervention assessments and signed parental consent and child assent forms were included in the analytic sample. Exclusions were due to missing or incomplete consent documentation, incomplete or illegible assessments, student absences on either pre‐ or post‐ (or both) assessment days, and logistical constraints at the school (e.g., scheduling disruptions or incomplete follow‐up). These exclusions were not based on student performance or teacher selection. Reliance on complete consent and assessment data introduces the potential for selection bias. Future evaluations should incorporate strategies to reduce missingness, such as streamlined consent procedures, digital assessments, or additional in‐class support.

Finally, while the off‐bike format enhances feasibility, equity, and scalability in school settings, it does not provide the same experiential learning opportunities as on‐bike instruction. Hybrid models that integrate scalable off‐bike education with targeted on‐bike components represent a promising direction for maximizing both accessibility and skill‐based learning.

### 4.3 Future Directions

Future research should extend beyond knowledge outcomes to examine additional dimensions of program effectiveness and implementation. Evaluations assessing behavioral skills—such as hazard recognition, decision‐making in simulated environments, or observed riding behaviors—would provide important insight into whether knowledge gains from the BESC translate into safer real‐world practices. Implementation‐focused research examining barriers and facilitators at the school and district levels, including scheduling constraints, teacher training needs, and administrative support, would further inform scalability and sustainability. Longitudinal studies assessing knowledge retention and skill development over time, as well as evaluations conducted in rural or suburban settings, would strengthen generalizability and guide broader dissemination of off‐bike bicycle safety education. Hybrid models that integrate scalable off‐bike instruction with targeted on‐bike experiential components represent a promising direction for maximizing both accessibility and skill‐based learning when resources permit.

## 5 Conclusions

The BESC improved bicycle safety knowledge among students in grades 2–5, with larger gains in younger students. The curriculum effectively increased understanding of critical safety concepts—helmet fit, visibility, predictability and built environment awareness. Unlike many programs that require bicycles, dedicated facilities, or in‐school cycling infrastructure, the BESC was designed for off‐bike, in P.E. settings with minimal equipment, making it highly scalable and accessible, particularly for under‐resourced schools. It is also one of the few programs tailored to developmental differences across elementary grade bands (grades 2–3, and 4–5), ensuring that safety concepts and active learning tasks are aligned with children's cognitive, motor, and perceptual capabilities. By integrating active learning, infrastructure literacy, and culturally responsive scenarios, the BESC addresses critical gaps in conventional bicycle education, including infrastructure navigation (e.g., bike lanes, shared‐use paths) and real‐world hazard recognition. Its low‐resource, off‐bike design positions it for broad integration into state and district P.E. curricula, advancing both equity and safety in youth mobility education.

## Funding

This work was supported by the Florida Department of Transportation through the Transportation Alternatives Program (G2E19). The funding source had no role in study design, data collection, analysis, interpretation, or manuscript preparation.

## Ethics Statement

The study was approved by the (University of Miami)'s Institutional Review Board (#20091106) on September 15, 2022.

## Consent

Informed consent was obtained from all subjects involved in the study.

## Conflicts of Interest

The authors declare no conflicts of interest. The BikeSafe Elementary School Curriculum evaluated in this study was developed and implemented by the University of Miami KiDZ Neuroscience Center as part of a grant‐funded injury prevention initiative. The authors received no personal financial benefit from the development, implementation, or evaluation of the curriculum.

## Supporting information


**Data S1:** josh70162‐sup‐0001‐Supinfo.pdf.

**Table S1:** School‐level demographic and socioeconomic characteristics of participating schools. School‐level demographic composition of participating schools, including student race/ethnicity, total enrollment, eligibility for free and reduced‐price lunch, and student sex distribution. These data provide contextual information on the socioeconomic and demographic diversity of the school populations included in the study.

## Data Availability

The data that support the findings of this study are available on request from the corresponding author. The data are not publicly available due to privacy or ethical restrictions.
