# Supplementary material for: Evaluation of a Curriculum‐Based Elementary School BikeSafe Program
Source: J Sch Health. 2026 May 5;96:e70162. doi: 10.1111/josh.70162 (PMC13143855; doi:10.1111/josh.70162)
Supplement: Supplementary file 1 — Data S1: josh70162‐sup‐0001‐Supinfo.pdf. [file JOSH-96-0-s002.pdf]

UHealth
UNIVERSITY OF MIAMI HEALTH SYSTEM
UNIVERSITY OF MIAMI
MILLER SCHOOL of MEDICINE

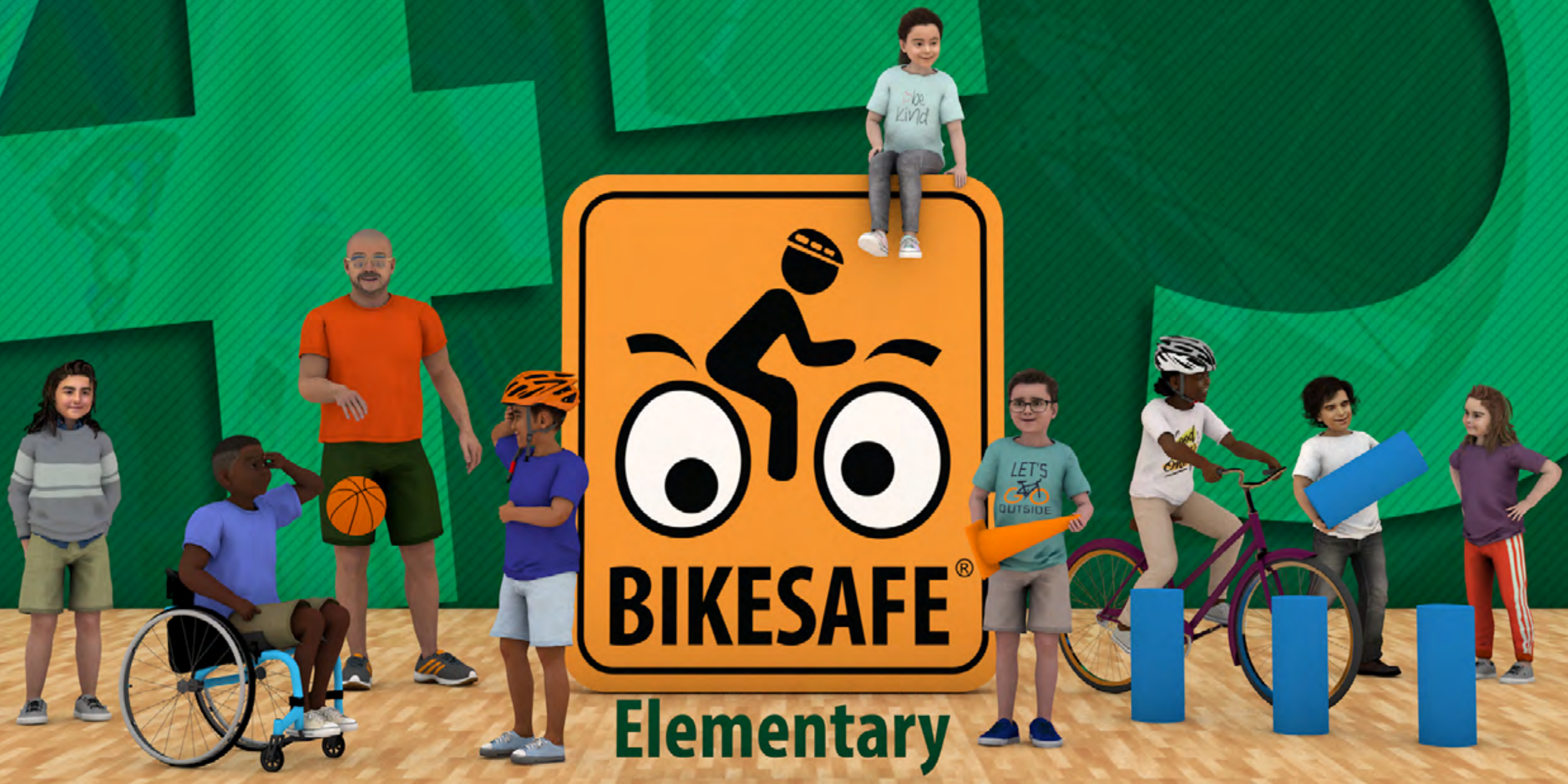

Physical Education Curriculum

Lessons & Activities - Grades 4 to 5

V1 | Print
07/30/2024

Program Director:
Dr. Gillian Hotz

Project Manager:
Dr. Michelina "Mickey" Witte

Content and activities:
Alyson Brady
Dr. Jayne D. Greenberg
Luke O'Neil
Grant Wentzel
Dr. Michelina "Mickey" Witte

Youth classroom educator:
Grant Wentzel, M.A.T.

Program Evaluation:
Dr. Valentina I. Kloosterman

Literature review:
Alyson Brady
Dr. Michelina "Mickey" Witte

Visual production:
Kurt Kaminer

The BikeSafe Program satisfies the national physical education standards set by SHAPE America: shapeamerica.org/standards/pe/

M-DCPS PE TEACHERS:

- Once you have implemented the curriculum in your PE classes for the school year, please submit a Curriculum Completion Form **- PER M-DCPS SCHOOL BOARD MANDATE -** at: **ibikesafe.org/ccf**

BIKESAFE Elementary

Table of Contents

- Introduction 6
- Before We Ride 8
- Signs & Signals 18
- Safe Places 30
- On-Bike Activities 42

About the BikeSafe Elementary 4-5 Curriculum:

Comments and Suggestions

For all inquiries, please contact us at:

bikesafe@miami.edu or **(305) 243-0349**

If your question or comment relates to this document, please provide the version number and publication date (bottom right corner of cover page), in order for us to provide you with accurate assistance.

Roles and Responsibilities

Users of the BikeSafe Curriculum and its related resources assume the responsibility of conveying the educational principles contained herein in a manner appropriately put forth by the curriculum text.

These resources do not contain every possible on-bike or off-bike scenario. It is important to ensure children are supervised by an adult at all times.

Teachers using this curriculum should first take the brief video training course at ibikesafe.org/schools/start **before** using it in the classroom.

It is the responsibility of the teacher to provide a safe educational environment.

Sharing This Curriculum

The content of the BikeSafe Curriculum, including all text and images, may not be extracted, repurposed, reused, or rebranded in any form by any other individual or entity without prior written permission from the University of Miami BikeSafe Program.

This document as published may not be distributed or uploaded anywhere other than the official BikeSafe webpage, ibikesafe.org

If you wish to share this document on your website, blog, social media, or elsewhere, please link to the following URL: ibikesafe.org/schools/start

Printed and digital copies of the BikeSafe Curriculum may be reproduced for classroom use in their original, unaltered forms.

Copyright

©2024 University of Miami BikeSafe Program. All rights reserved.

The University of Miami Miller School of Medicine ("University") nor any of its officers, trustees, agents, employees, faculty, students, volunteers or the BikeSafe program makes any warranty, express or implied, about the accuracy or completeness of any information contained in this publication. The University makes no representations or warranties about use of the BikeSafe program. Accordingly, you acknowledge and agree that you shall use the program at your own risk and disclose to participants that their use is at their own risk. The UNIVERSITY, its officers, trustees, agents, employees, faculty, students, volunteers and the BikeSafe program disclaim all responsibility for information contained in this document and all liability (including without limitation, liability in negligence) for all expenses, losses, damages and costs you may incur as a result of the information being inaccurate or incomplete in any way or for any reason. The UNIVERSITY, its officers, trustees, agents, employees, faculty, students, volunteers and the BikeSafe program disclaim all liability for any damages arising from your access to or use of any material or part thereof within this publication and all related documents, and do not assume any legal liability or responsibility for the accuracy, completeness, or usefulness of information, apparatus, product, or process disclosed; nor represent that its use would not infringe privately owned rights.

Curriculum Overview

Thank you for choosing the latest University of Miami BikeSafe Physical Education Curriculum for elementary schools.

This curriculum has been created with new and unique learning modules, each of which contain off-bike physical activities. The activities are age-appropriate and require minimal equipment, enabling students to apply the learning objectives in a physically active, creative manner.

Feedback is always appreciated. It can be provided either through the BikeSafe Curriculum Completion Form (required by Miami-Dade Public Schools and found at ibikesafe.org/ccf) or through email (bikesafe@miami.edu).

Even if you are familiar with earlier publications of the BikeSafe Curriculum, we recommend reading through this edition entirety, due to the changes in both content and narrative.

What's Included?

The BikeSafe Elementary Curriculum is divided into individual modules, each building upon the other. As such, each module is designed to be taught in consecutive order (Module 1, then Module 2 and so on) in their entirety.

The document that you are reading now contains everything a physical education instructor will need to effectively teach the curriculum.

While safety equipment and safe behavior are important, neither are a guarantee that people will have safe experiences on roads designed for cars. This is why BikeSafe emphasizes that connected networks of protected bike lanes, paths and trails are **necessary** for the safety of families, youths, and risk-averse riders.

Please remember that children must always be supervised by an adult, even while riding on safe bicycle infrastructure.

Recommended Implementation

In school districts where the BikeSafe Curriculum is mandated - such as Miami-Dade - your district may recommend the curriculum to be taught at a specific time (or times) during the school year.

PE teachers from each school must submit a Curriculum Completion Form (CCF) each school year to the University of Miami, through the BikeSafe website.

The CCF is available at: ibikesafe.org/ccf.

For educators using the BikeSafe Curriculum where no formal agreement exists with their school board and the University of Miami, these modules can be taught at any time during the school year. However, keep in mind that the modules are designed to be taught sequentially.

Whether or not your school district mandates the implementation of the curriculum, it is always helpful to submit a CCF. It helps us track usage and hear your feedback.

Accommodations

Instructors may provide an alternative, adaptive learning plan for exceptional students. They may set unique goals allowing them to gesture and/or recall two to three of the vocabulary terms and/or key concepts in accordance with their learning style and their Individualized Educational Plan (IEP).

Feedback? Yes, please!

If you have any comments or feedback regarding the BikeSafe Curriculum, please reach out to us. Our office hours are Monday through Friday, 9-5pm EST.

Office phone: 305-243-0349

Email: bikesafe@miami.edu

Mailing address: University of Miami BikeSafe Program
KiDZ Neuroscience Center
1095 NW 14th Terrace, Suite 1-26
Miami, Florida 33136

Introduction

What is bicycle safety?

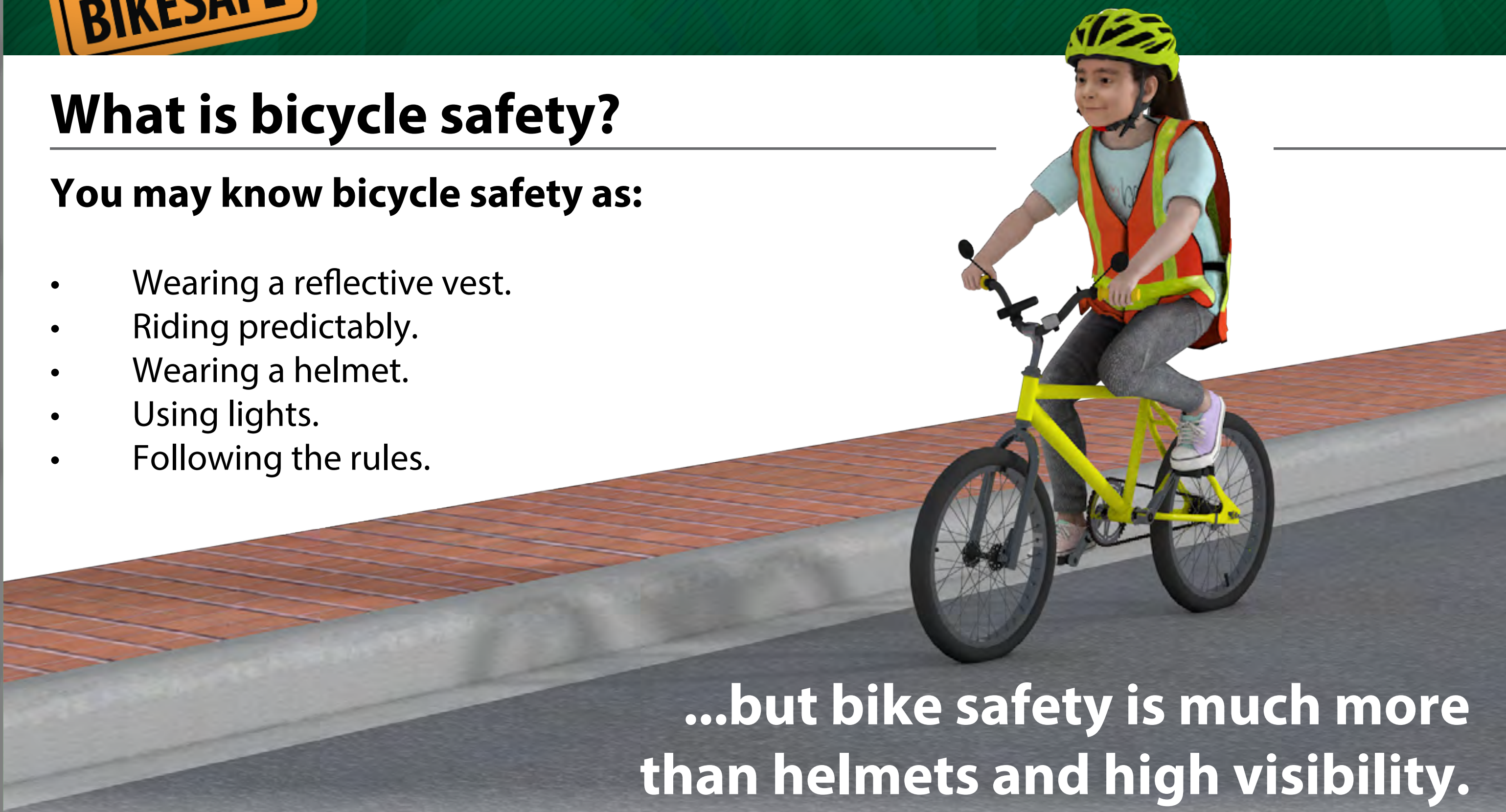

You may know bicycle safety as:

- Wearing a reflective vest.
- Riding predictably.
- Wearing a helmet.
- Using lights.
- Following the rules.

...but bike safety is much more than helmets and high visibility.

Bicycle safety is also:

- A fun and positive experience.
- A direct and convenient route.
- A place where children can ride in safety.
- A protected space where mistakes aren't life-threatening.

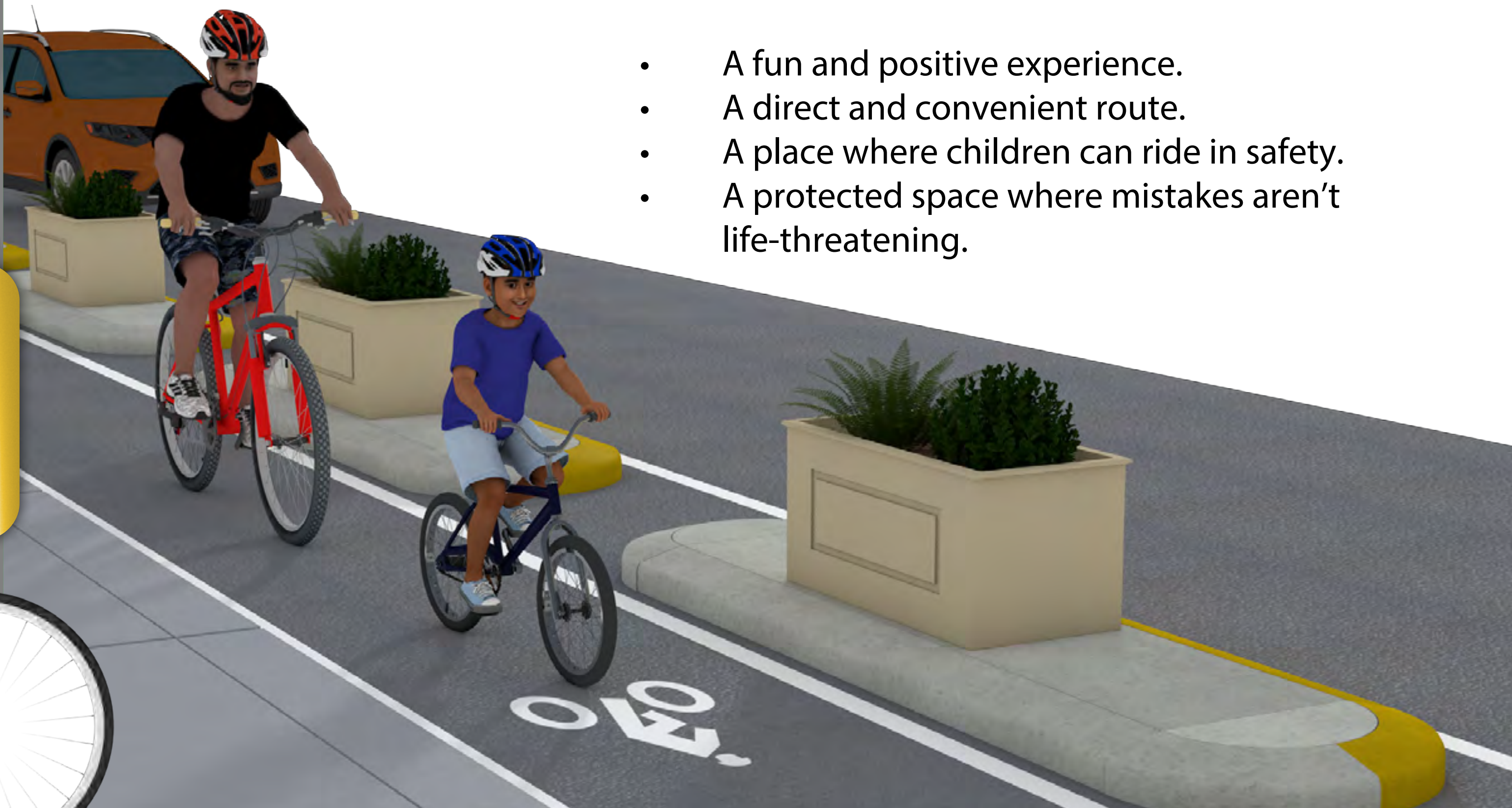

While helmets, lights, and predictability can enhance safety, safe places - such as protected bike lanes, protected intersections, and dedicated bicycle signals - provide exceptional safety benefits to riders of all ages.

This curriculum will help you teach your students the elements of traditional bicycle safety, in addition to the latest information about protected bike lanes and other safe places.

TEACHING TIP

This introduction is intended for PE teachers, but may be used to supplement class Q&A sessions.

Elementary

Before We Ride

Before We Ride: Introduction

Safety equipment can help increase visibility. This module explains the difference between reflectors and lights and how hand signals help convey that a rider is turning or stopping.

Key vocabulary:

- **Visibility**
- **Bicycle lights**
- **Reflectors**
- **Hand Signals**

Time to complete:

- **~30 minutes**

Learning Objectives

In this module, students will learn about:

- The importance of **visibility**
- Using **bicycle lights** and **reflectors** to *improve* visibility
- Using **hand signals** to indicate direction to others

Teacher Script - Q&A

(5-10 minutes)

Visibility

Q. "What is **visibility**?"

A. "**Visibility** is how easy someone else can see another object, such as a bicycle rider, especially at dusk, dawn, or night."

Bike Lights

Q. "What is the difference between a **reflector** and a **bicycle light**?"

A. "A **reflector** bounces a beam of light from another source, while a **bicycle light** emits a light source on its own.

Both can be used to improve **visibility**. Lights can be used to improve **visibility** in daytime too."

Hand Signals

Q. “What are **hand signals** for?”

A. “**Hand signals** let others know which way we are going when we stop, turn, or slow down.”

TEACHING TIP

While reflectors are frequently used on clothing, the standard reflectors fitted to all new bicycles may provide a false sense of security.

Because reflectors rely on another light source, they’re not always easy to see, and may not be illuminated at all from some angles.

Lights are more likely to be seen and may be required while riding at night - check your state and local laws.

Instructors are encouraged to use the following images with their class for demonstration (pages 10 through 15).

No lights

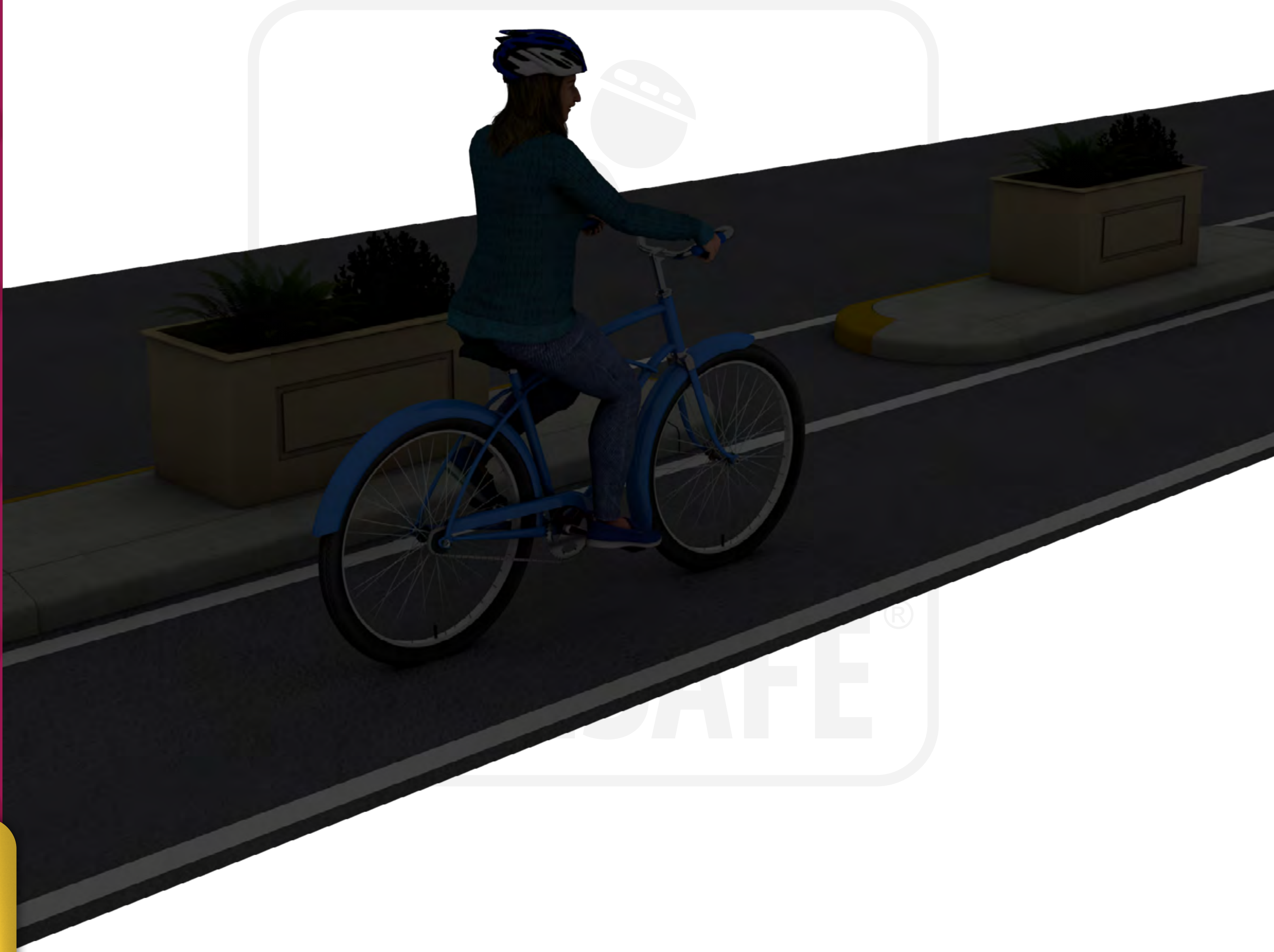

Reflectors

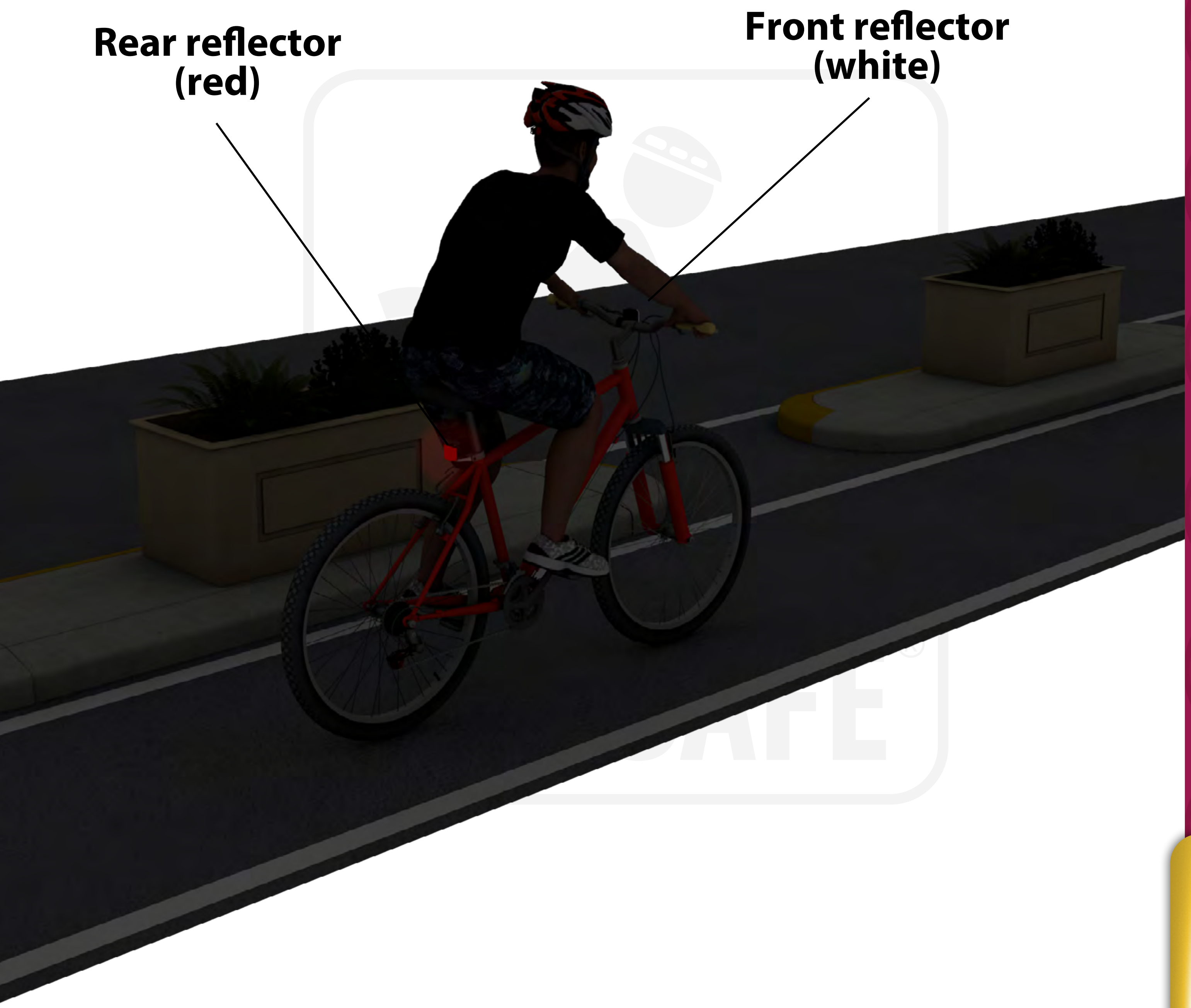

Lights

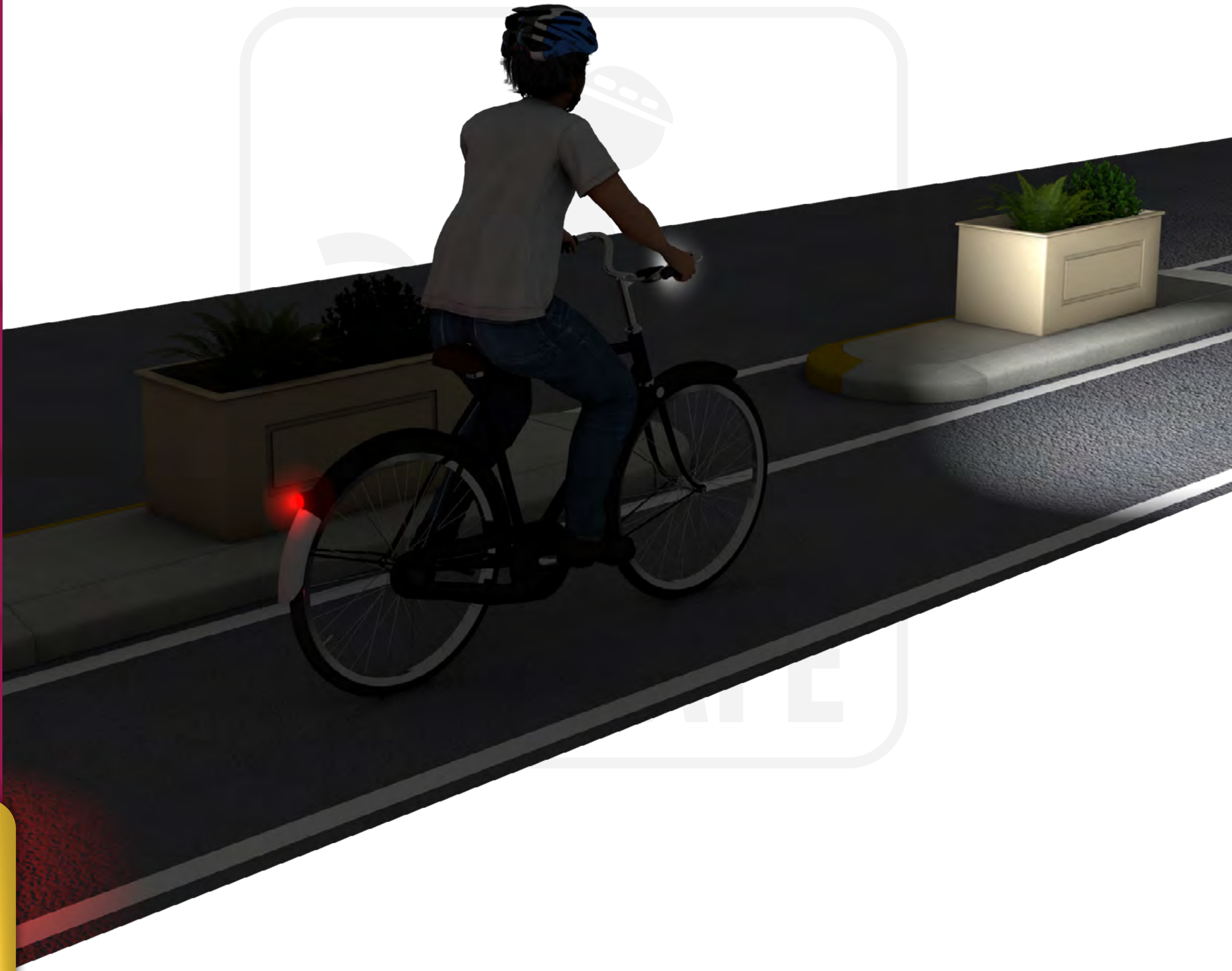

Left turn

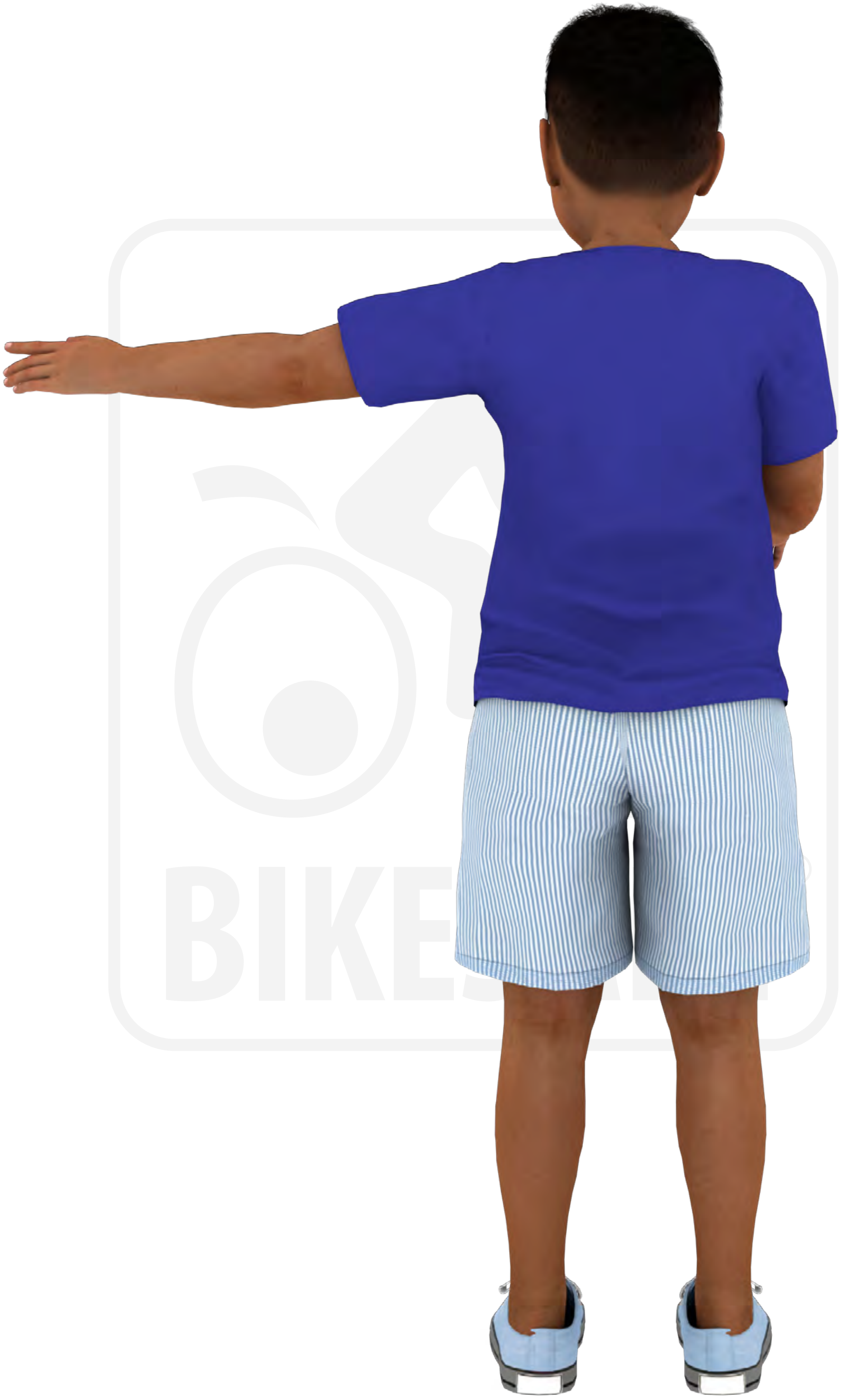

Right turn

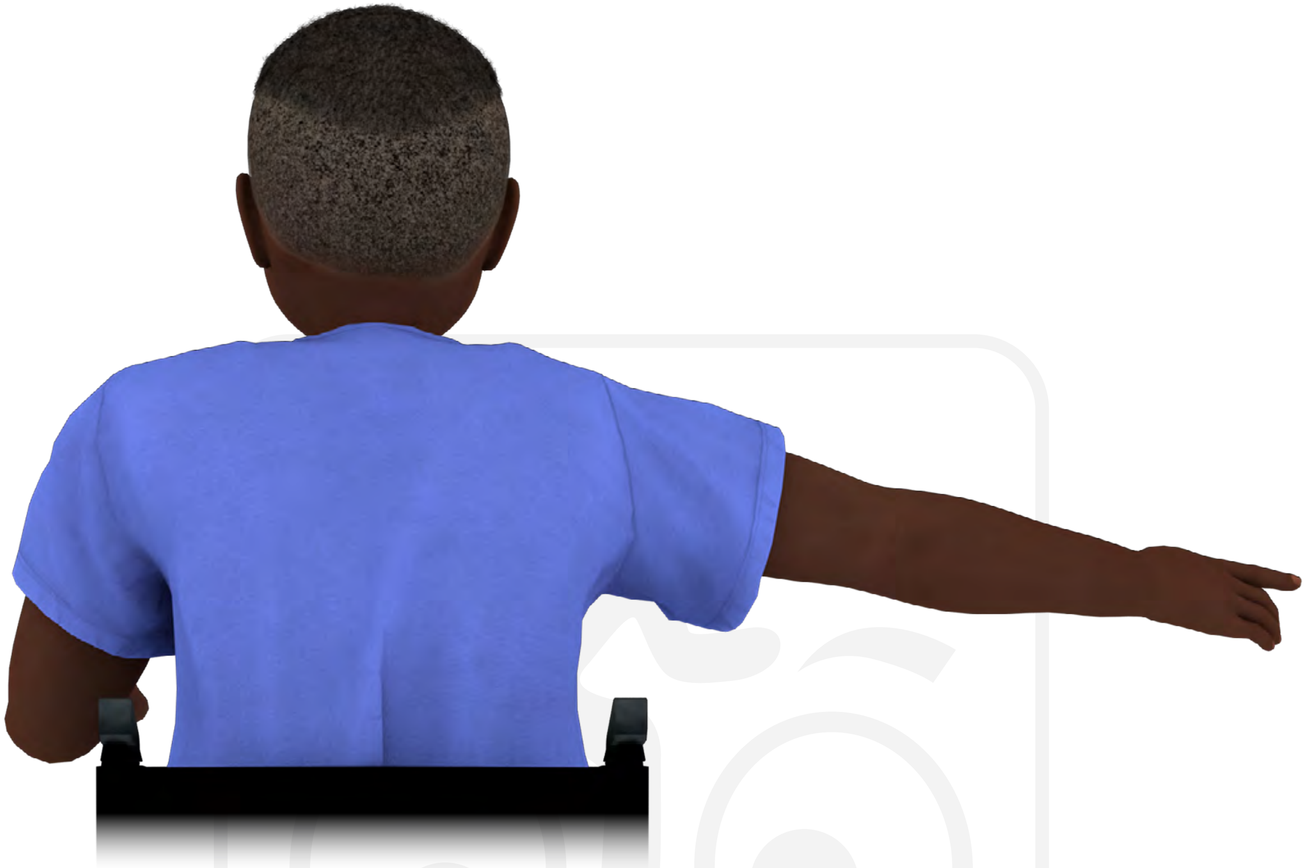

Alternate right turn

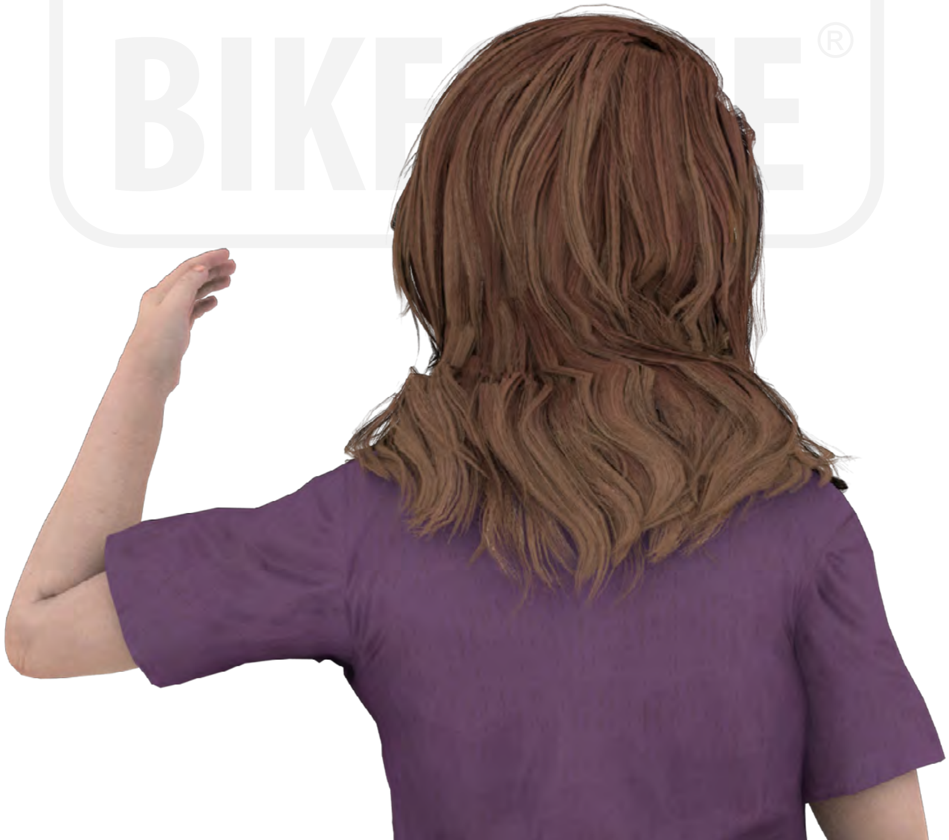

Stopping or slowing

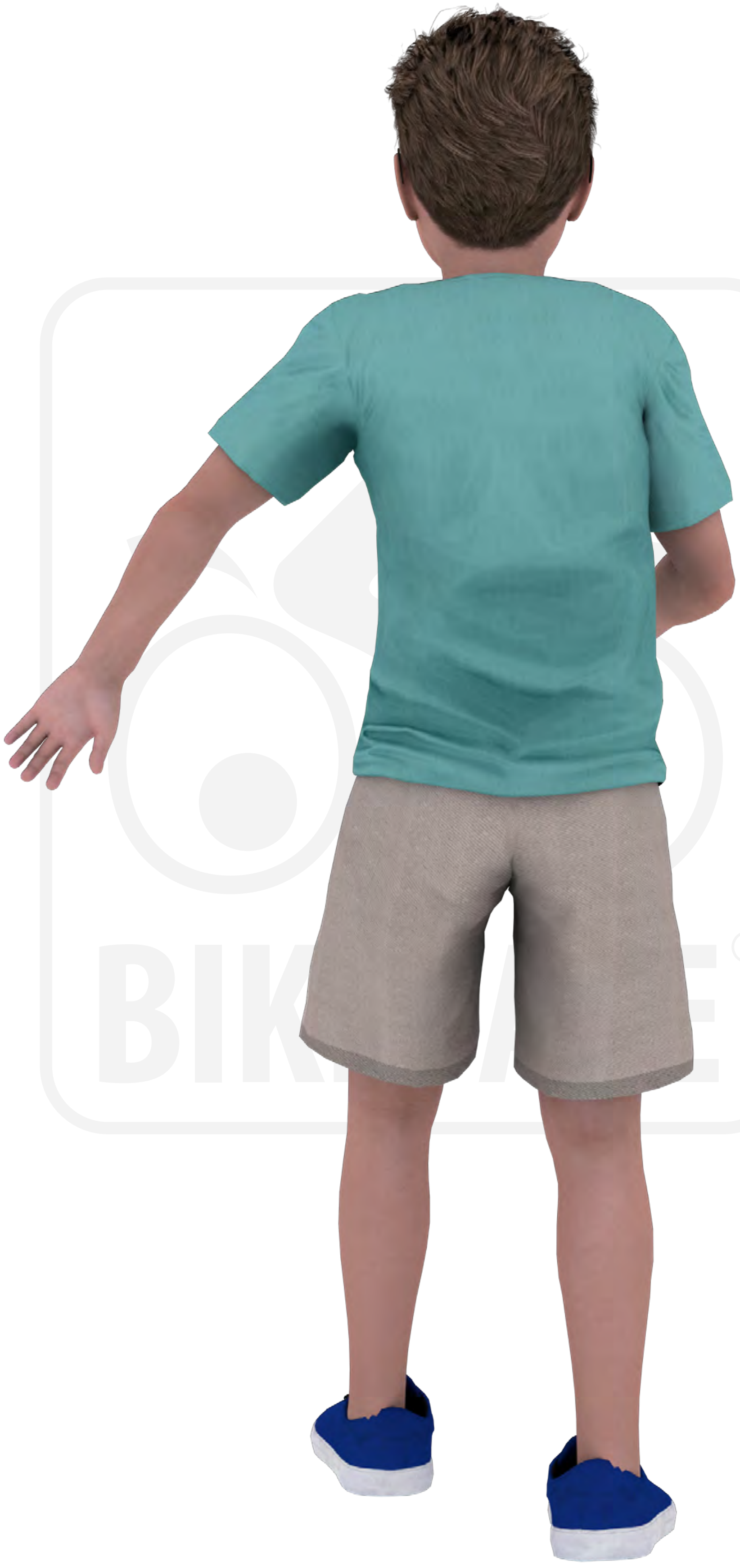

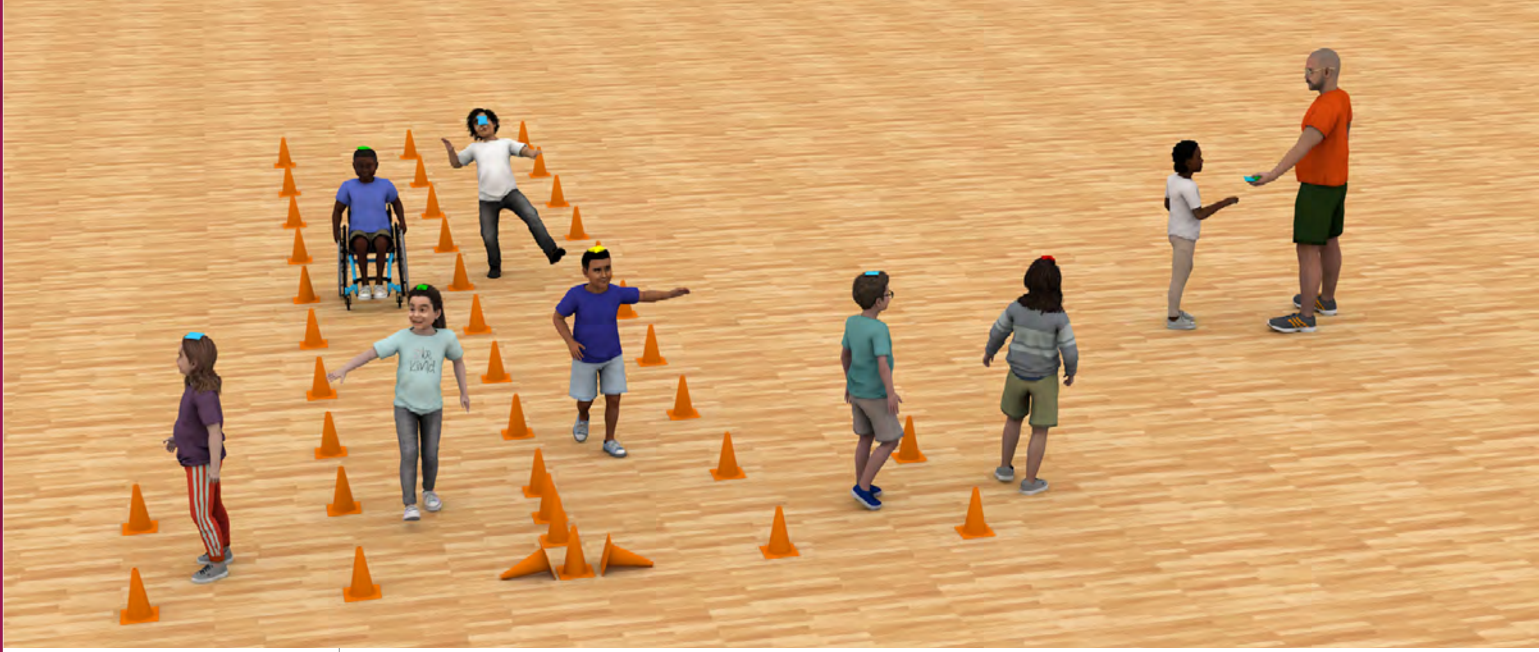

Activity - Bean Bag Balance

(15 minutes)

*Students will demonstrate the use of proper **hand signals** in this activity.*

To play:

1. Before you begin, create "traffic lanes" with cones or other PE props. Each lane should include a left or right turn.

2. Provide each student with a bean bag. Instruct them to place the bean bag on their head. Emphasize that balancing the bean bag is more important than speed.

3. Instruct students to walk inside the lanes while balancing the bag on their heads. When they get to the turning area, they must stop and use the correct **hand signal** before turning in that direction. Repeat as desired.

Optional:

- Floor scooters can be used for this activity, if available.
- This activity can be played as a relay race with older students.

Module Closure

(5 minutes)

Please remind your students of these key concepts before dismissing class:

- **Lights** and **reflectors** can improve **visibility**, making a rider easier to see. **Lights** can be used in the daytime too.
- **Hand signals** let others know in what direction we are going, especially when turning.

TEACHING TIP

Allow time for student questions during closure.

Instructor Notes

Signs & Signals: Introduction

Traffic signs, lights and signals create order by indicating when to cross an intersection. This module discusses how to use these signals effectively and the safety that "No Turn on Red" signs provide.

Key vocabulary:

- **Traffic Signs**
- **Traffic Lights**
- **Predictable**
- **No Turn on Red**

Time to complete:

- **~30 minutes**

Learning Objectives

In this module, students will learn:

- Basic **traffic signs** and **traffic lights**
- How **predictable** behavior enhances safety
- The importance of **"No Turn on Red"**

Teacher Script - Q&A

(5-10 minutes)

Traffic Signs and Lights

Q. "What is the purpose of **traffic signs** and **traffic lights**?"

A. "**Traffic signs** and **traffic lights** direct traffic, instructing us when to go and when to wait."

Traffic signs:

Stop sign	=	"Stop."
Yield sign	=	"Let others go first."

Traffic lights:

Green	=	"Go."
Yellow	=	"Slow down, prepare to stop," or "caution."
Red	=	"Stop."

TEACHING TIP

Remind students that it is best to cross with a parent or trusted adult.

Predictable Behavior

Q. "Who can tell me why **predictable** behavior is important for safety?"

A. "**Predictable** behavior makes it easier for everyone to understand when to stop, wait, and go.

Traffic signals and **hand signals**, for instance, help increase **predictability**."

No Turn on Red

Q. "What is a '**No Turn on Red**' sign?"

A. "Some intersections have a **No Turn on Red** sign. This sign tells drivers they cannot make a right turn at a red light.

No Turn on Red signs are an important enhancement for safety. This is because when drivers turn on red, they are unpredictable.

Drivers turning on red often do not see people walking or riding at intersections, putting riders - and pedestrians - in danger."

Stop sign

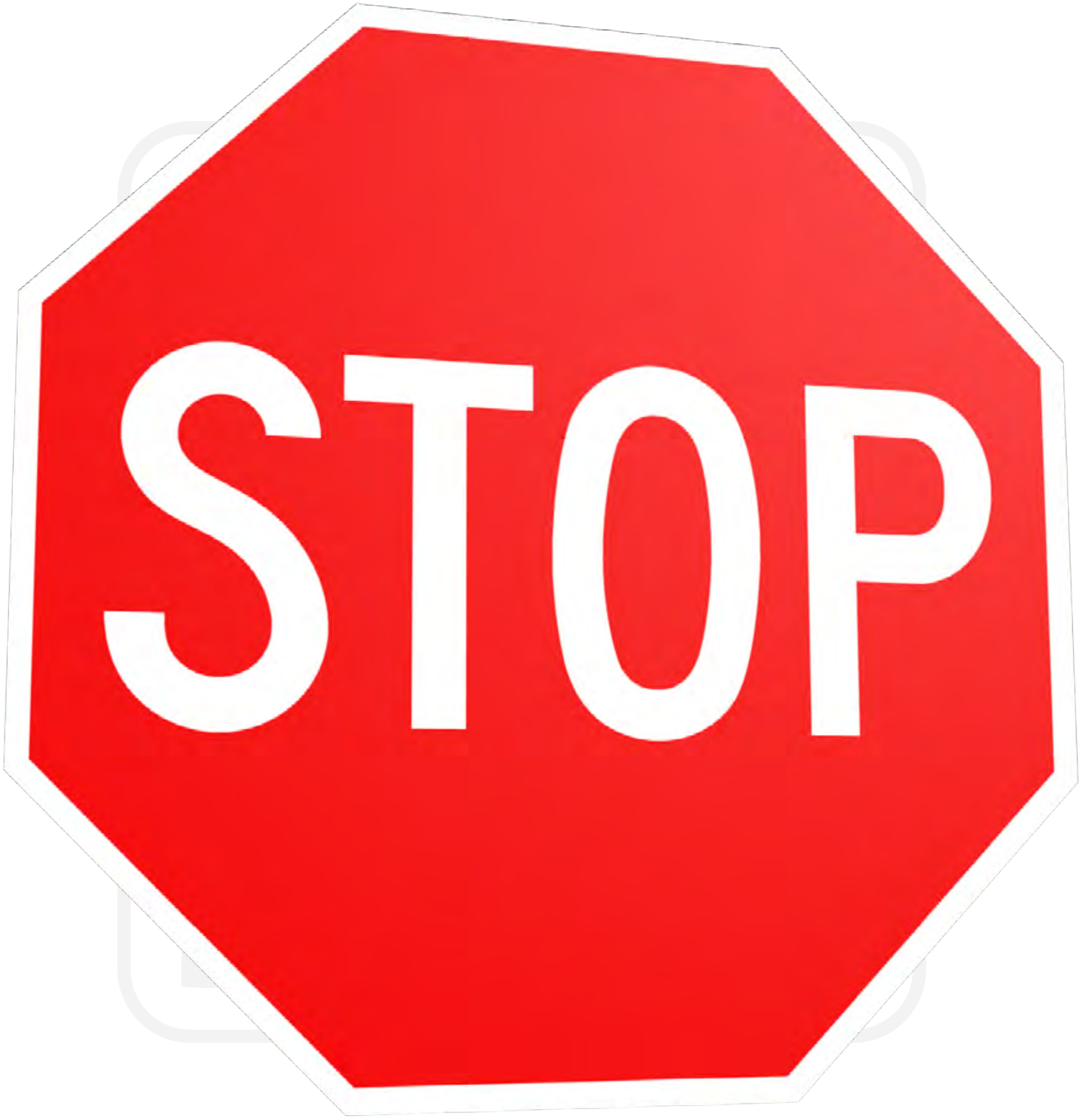

Yield sign

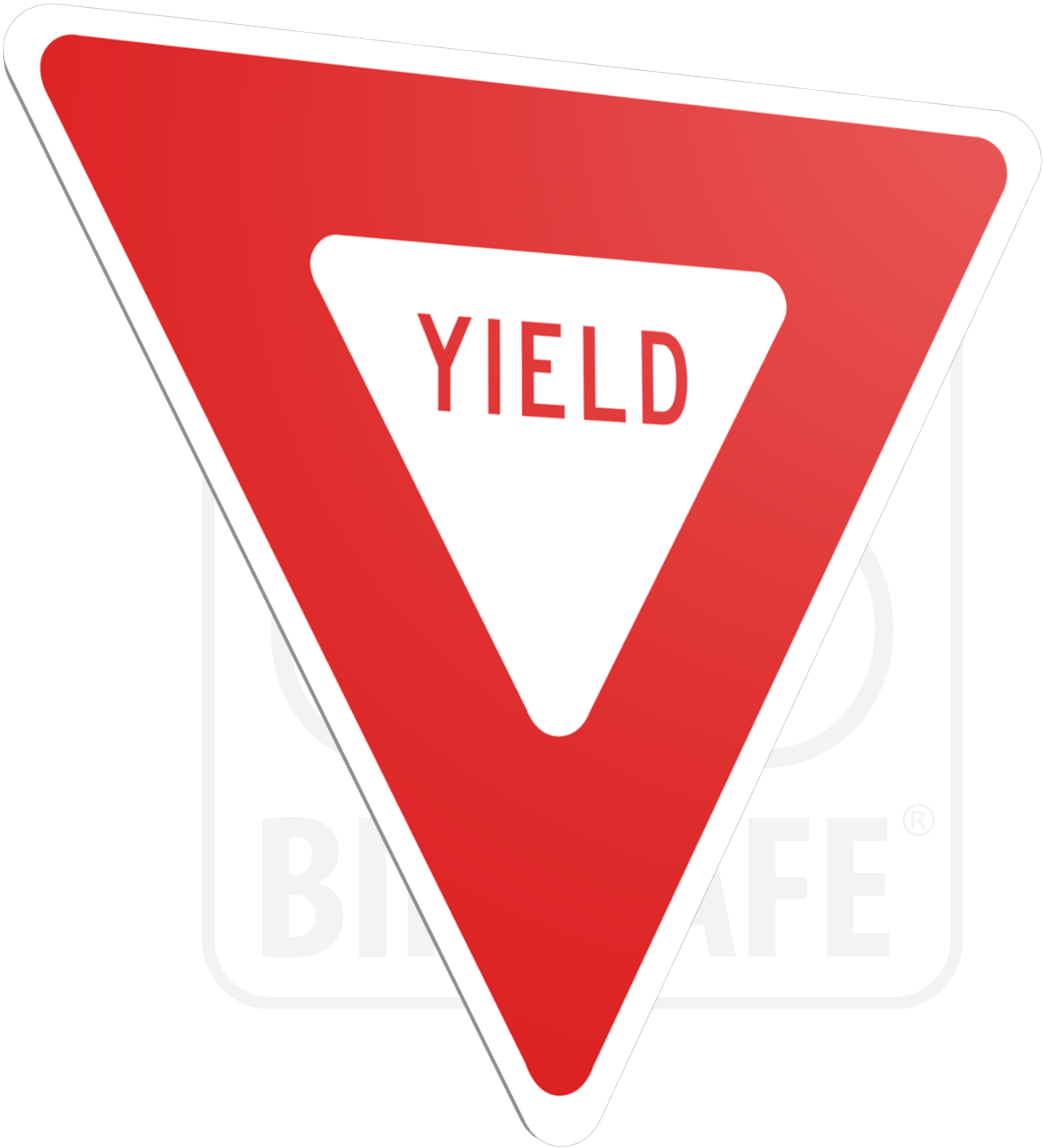

Green = “Go”

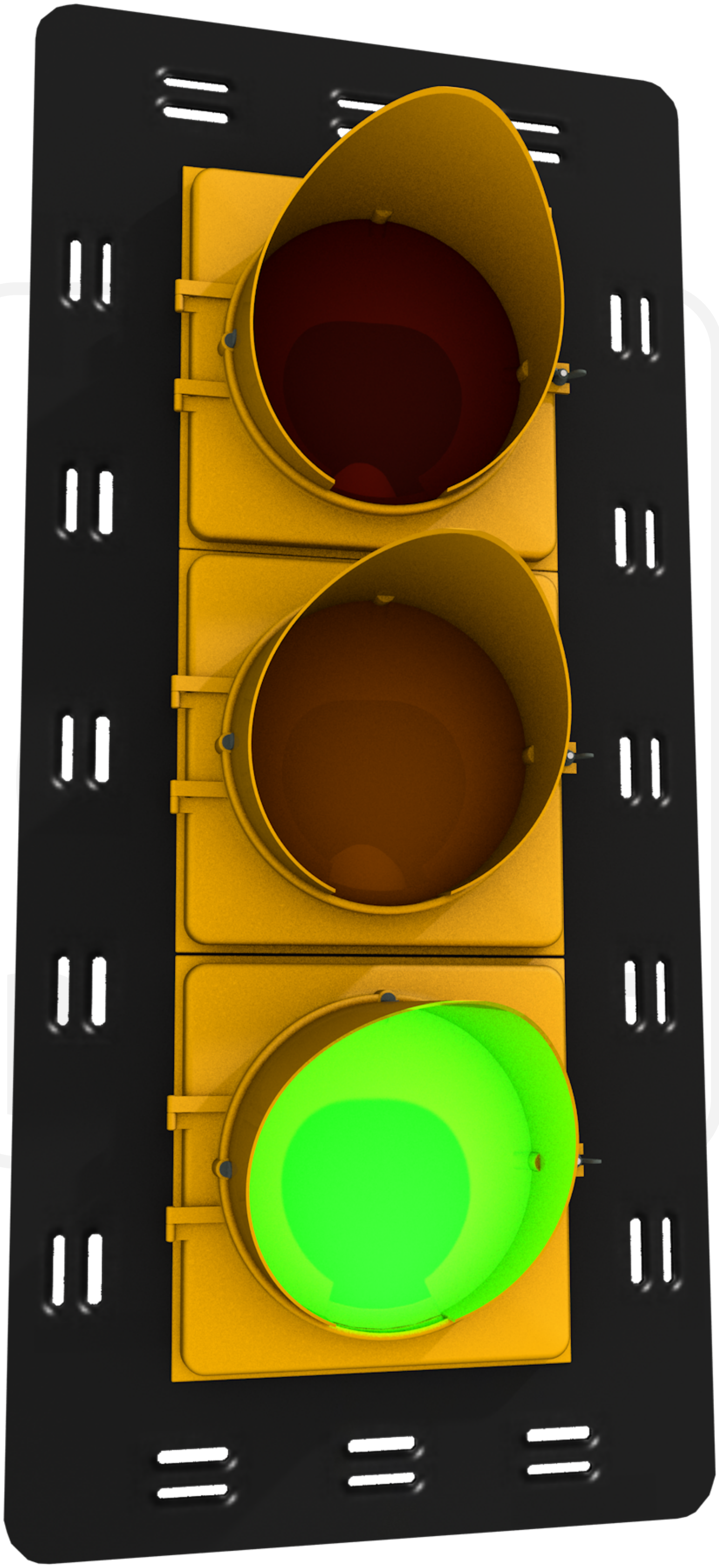

Yellow = “Slow Down”

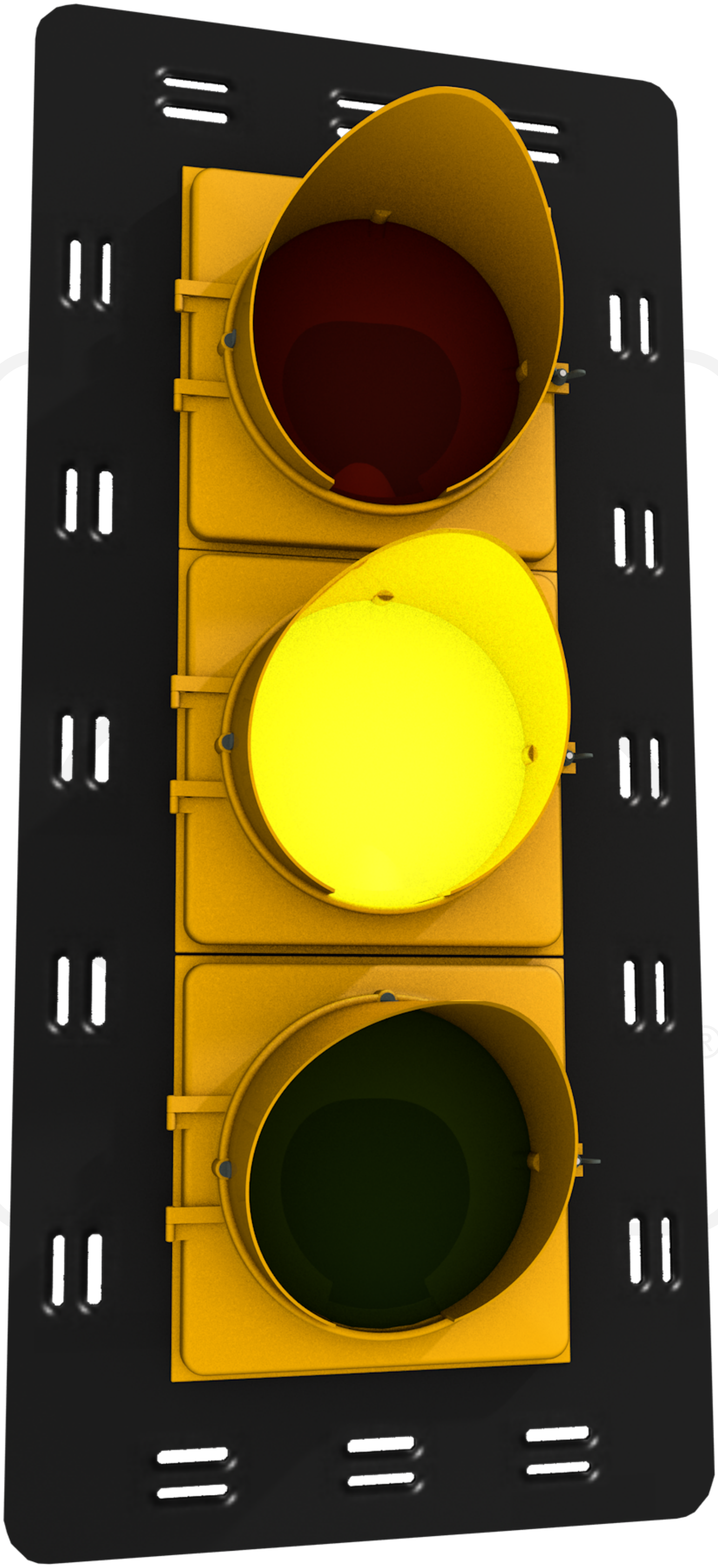

Red = "Stop"

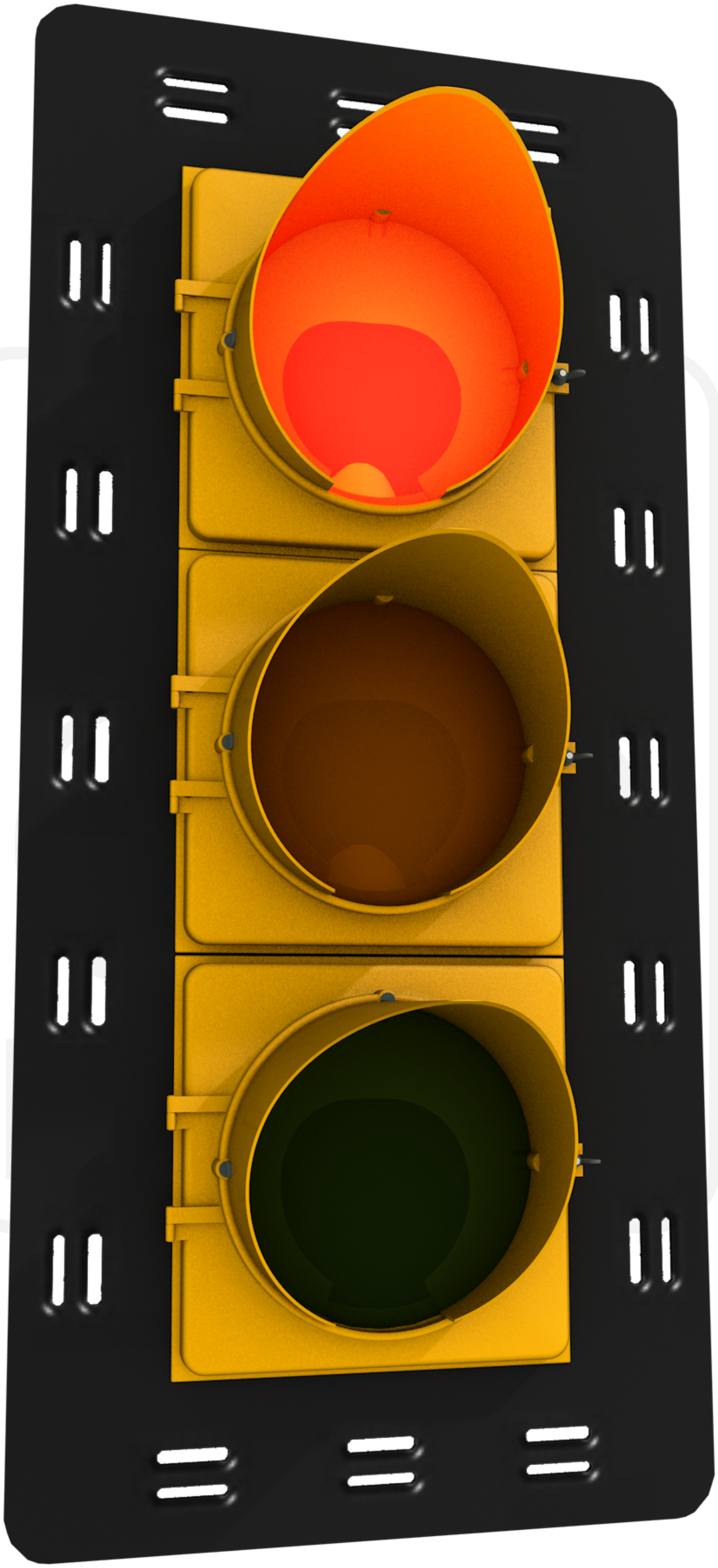

Driver turning on red

Dangerous for pedestrians

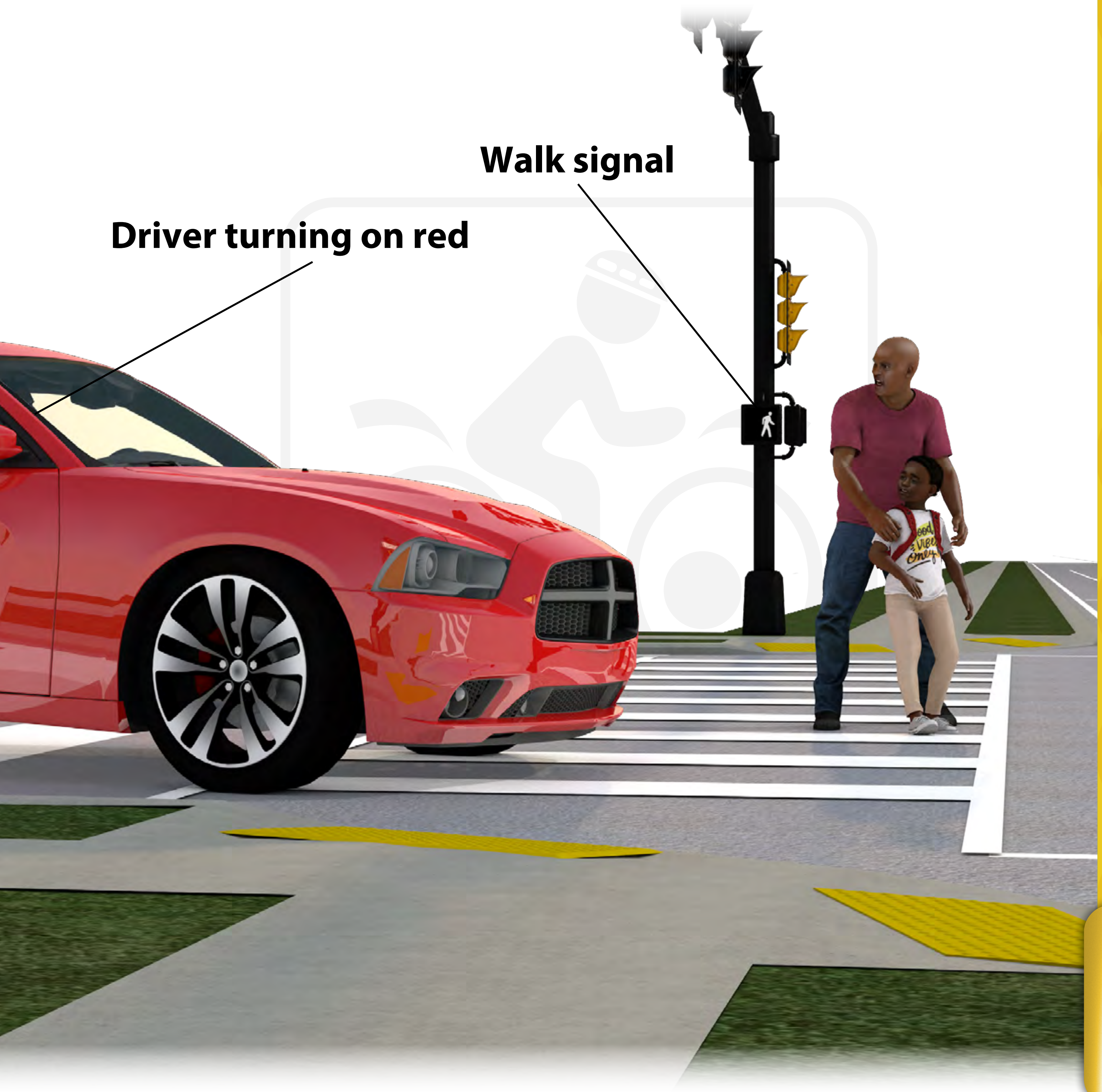

Driver turning on red

Dangerous for riders

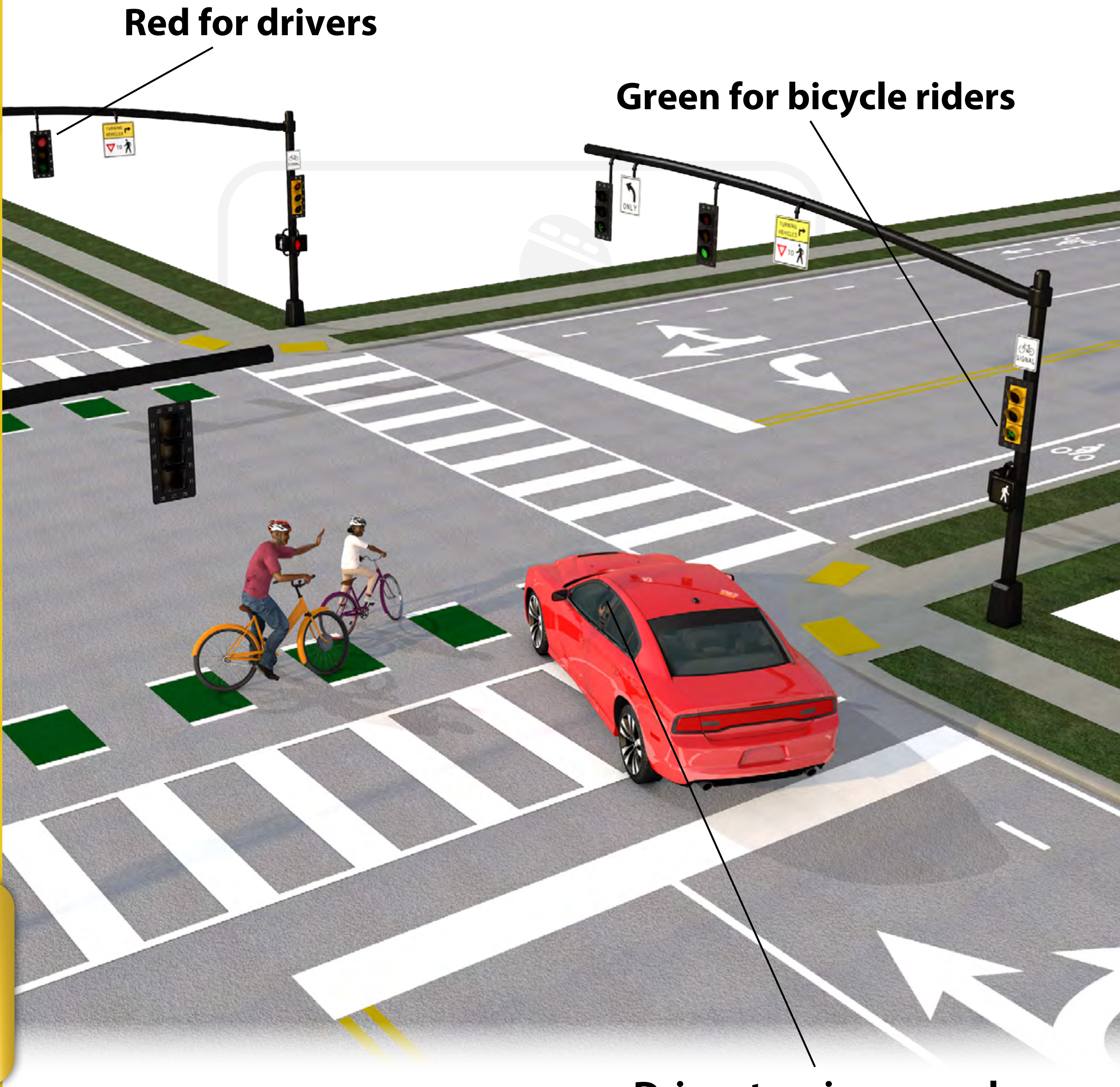

No turn on red

Safer for everyone

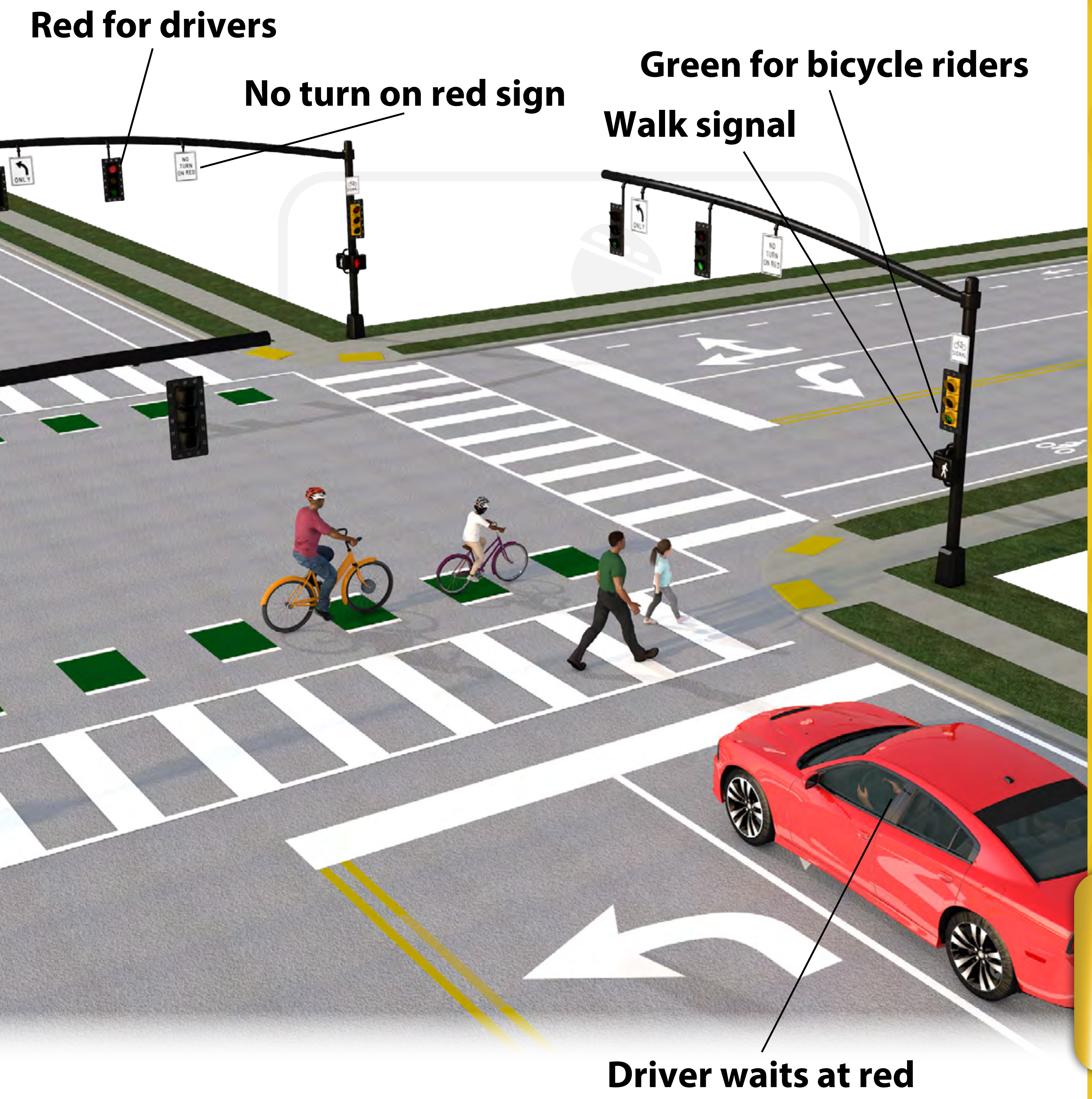

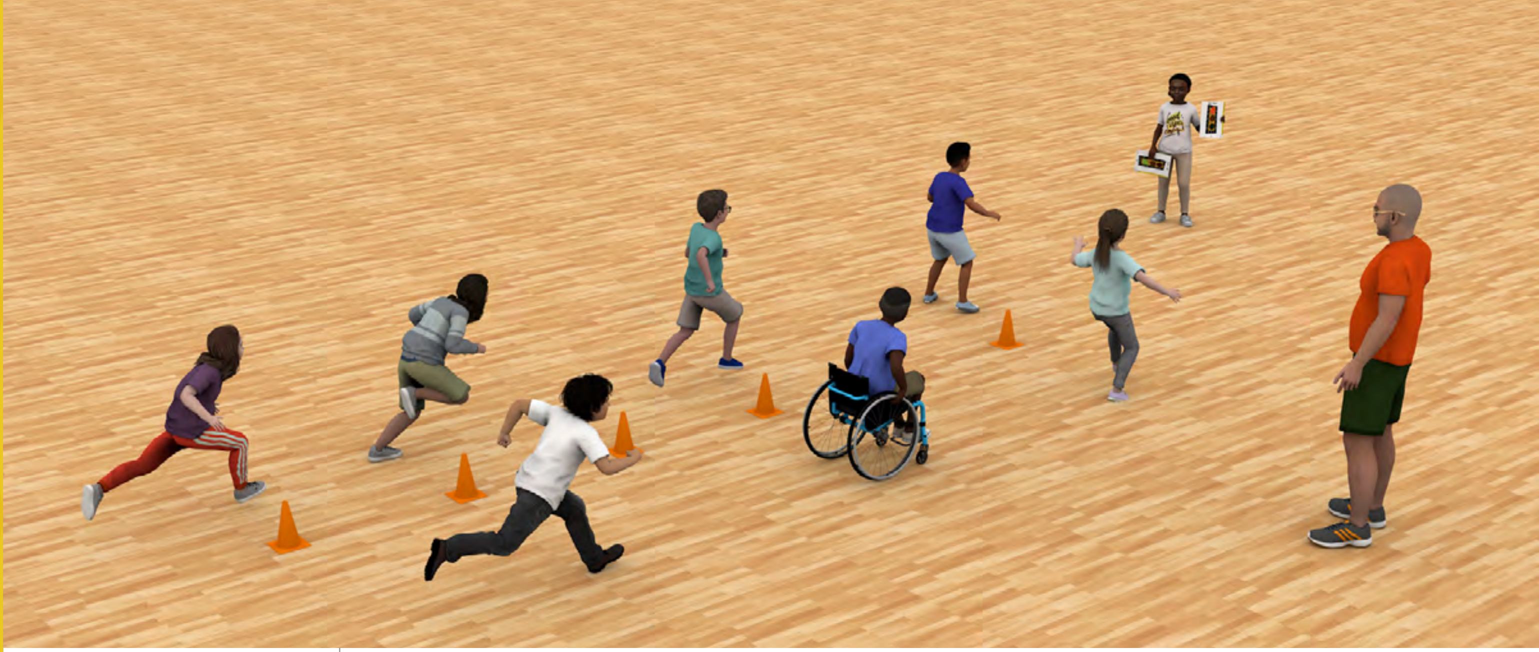

Activity - Red Light / Green Light

(15 minutes)

*In this activity, students will follow **traffic signals**, just as when they ride their bicycles.*

To play:

1. Line up students in rows at one end of the court or field.

2. Instruct one student to stand at the opposite end to call out signal colors:

"Green Light!"	=	Walk forward.
"Yellow Light!"	=	Move in slow motion.
"Red Light!"	=	Stop.

3. If a student doesn't slow down on yellow or keeps moving on red, they are sent back to the starting line. Rotate students until class time is over.

Optional:

- Consider printing copies of the red, yellow, and green **traffic signals** from pages 22 through 24 for this activity.
- For larger classes, consider creating two rows of students in an X shape, forming an intersection. The correct traffic lights must be held up for each direction of traffic to prevent crashes.

Module Closure
(5 minutes)

Please remind your students of these key concepts before dismissing class:

- **Traffic signs** and **traffic lights** indicate when to go and when to stop. They are for everyone using the road.
- **"No Turn on Red"** signs make intersections safer by preventing drivers from entering crosswalks and/or bike lanes at the wrong time.

Instructor Notes

Safe Places: Introduction

Key vocabulary:

- **Protected Bike Lane**
- **Safe Streets**
- **Traffic Calming**

Time to complete:

- **~30 minutes**

Street design greatly affects safety. This module explains how bike lanes with barrier protection create a safer, more inviting place for riders of all ages and how traffic calming provides further safety enhancements.

Learning Objectives

In this module, students will learn to:

- Identify types of bike lanes, including **protected bike lanes**
- Understand safe places to ride, such as **safe streets**
- Explain the benefits of **traffic calming**

Teacher Script - Q&A

(5-10 minutes)

Protected Bike Lanes

Q. "What is a **protected bike lane**?"

A. "A **protected bike lane** is a bike lane that has physical barriers, such as concrete curbs, separators, or planters. These barriers protect bicycle riders from drivers.

TEACHING TIP

Remind students that not all bike lanes are the same.

Protected bike lanes are safest because physical barriers separate and protect riders from drivers.

Painted bike lanes with no physical barriers or unprotected, green bike lanes are not as safe as protected bike lanes.

Safe Streets

Q. "What is a **safe street**?"

A. "A **safe street** is designed so people can walk, roll, or ride on it without getting hurt.

Safe streets have slower speeds and **traffic calming** that help improve our safety."

Traffic Calming

Q. "What does '**traffic calming'** mean? What ways can we calm traffic?"

A. "**Traffic calming** are measures to slow drivers down in areas where people are riding, walking, or rolling.

Protected bike lanes are a form of traffic calming. So are speed humps, raised crosswalks, and dedicated bicycle boulevards that filter traffic."

TEACHING TIP

Recommend that students ride with an adult until at least 10 years of age.

Basic bike lane

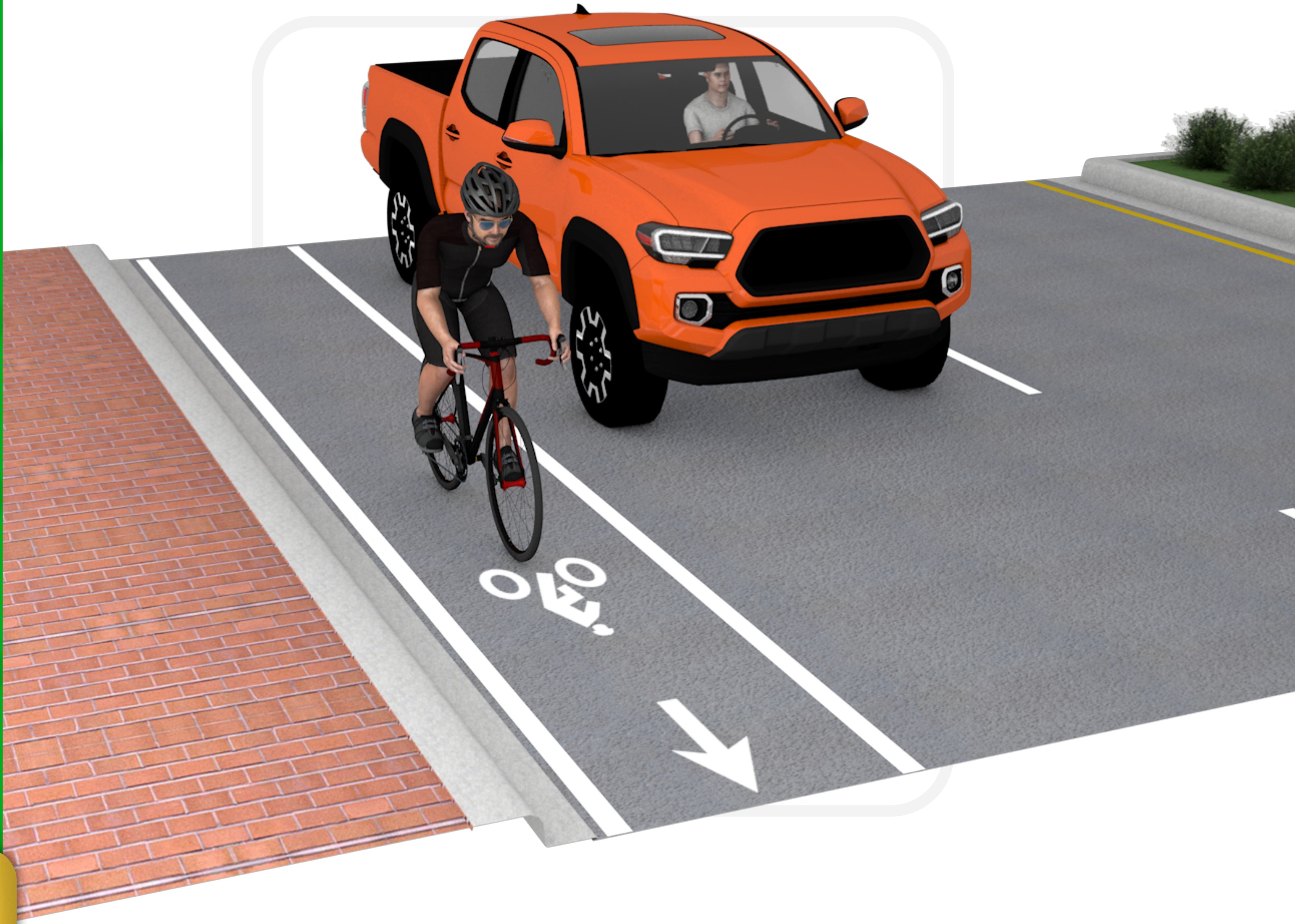

Green bike lane

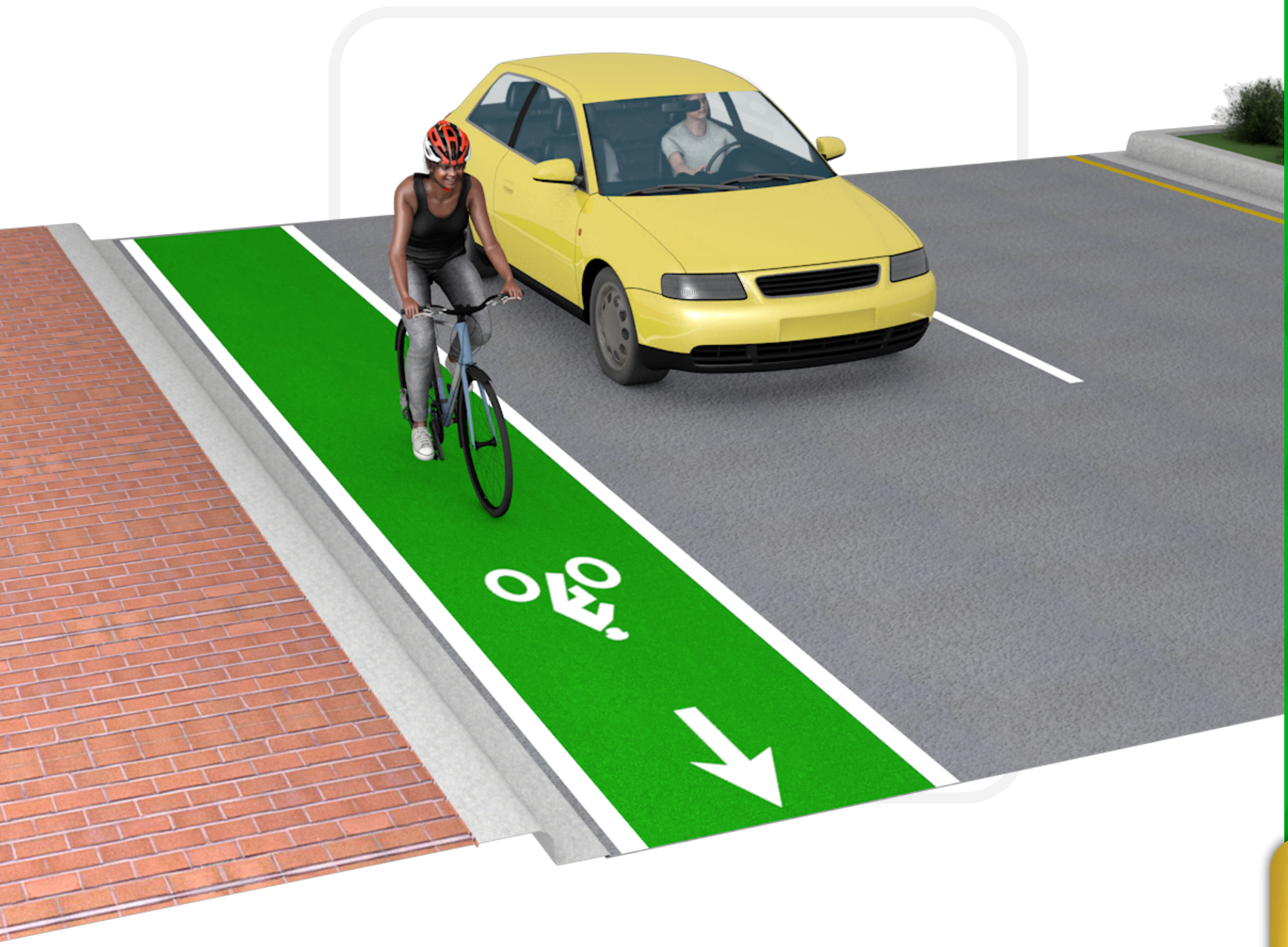

Protected bike lane

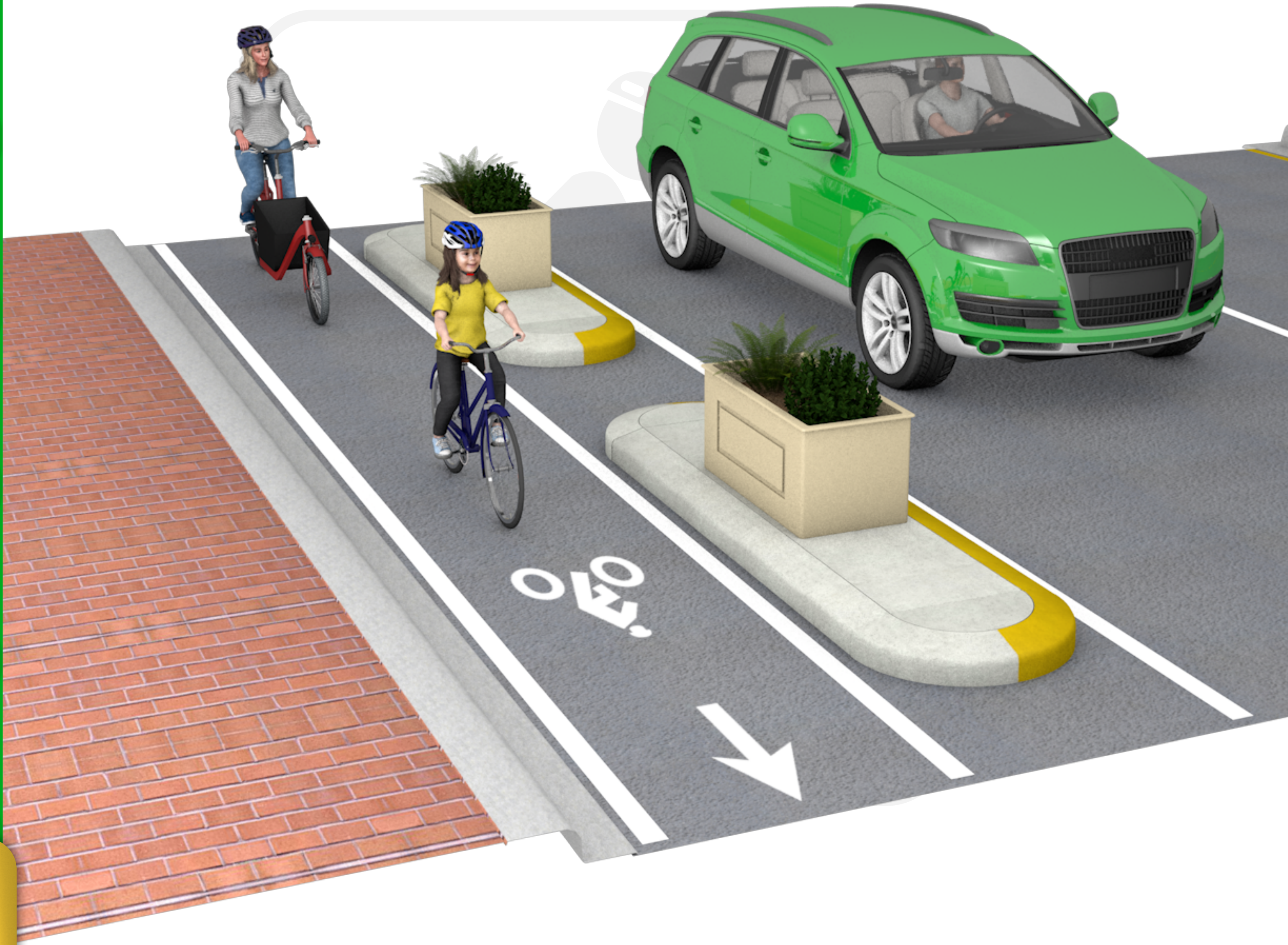

A Safe Street

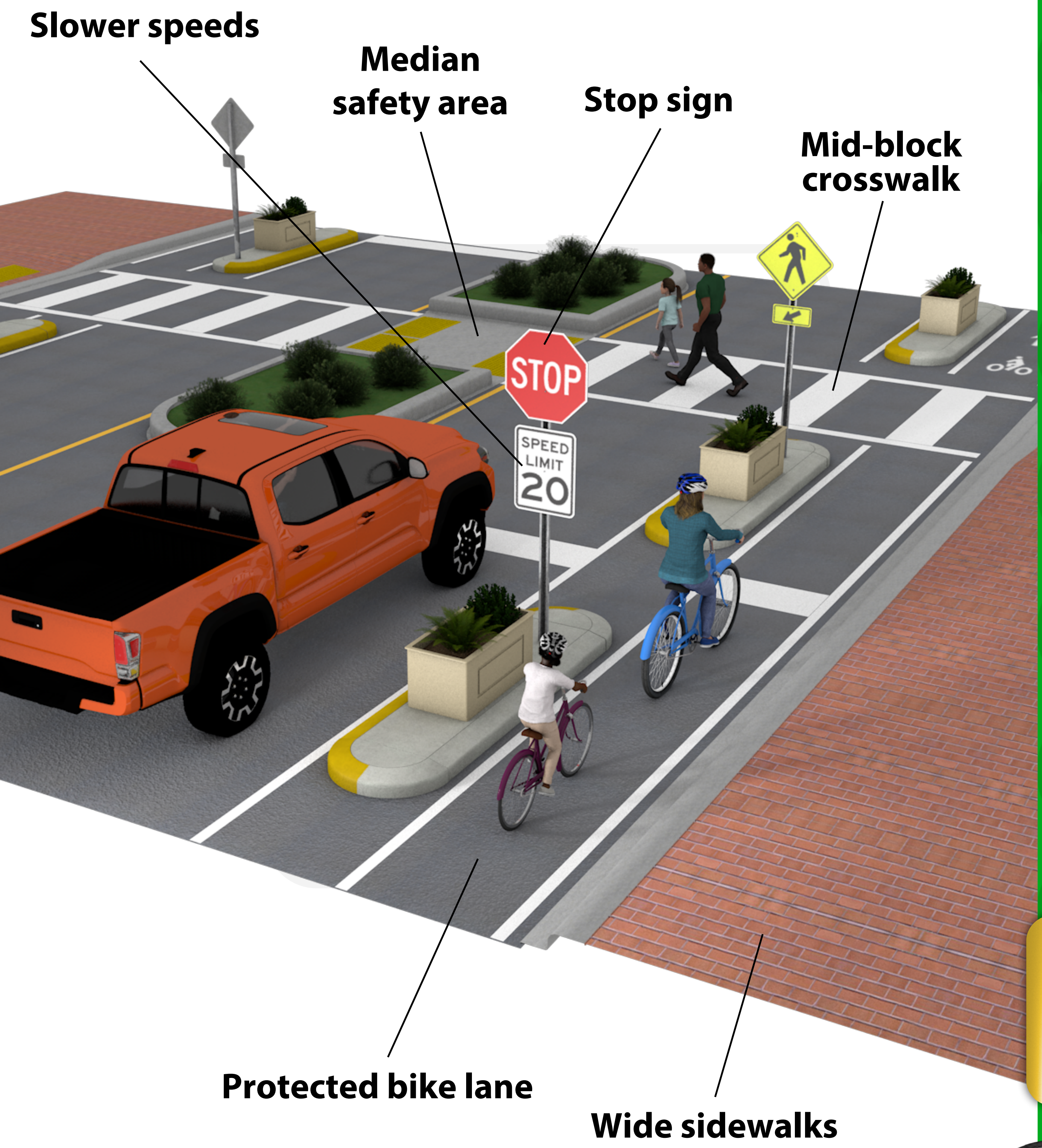

Speed hump

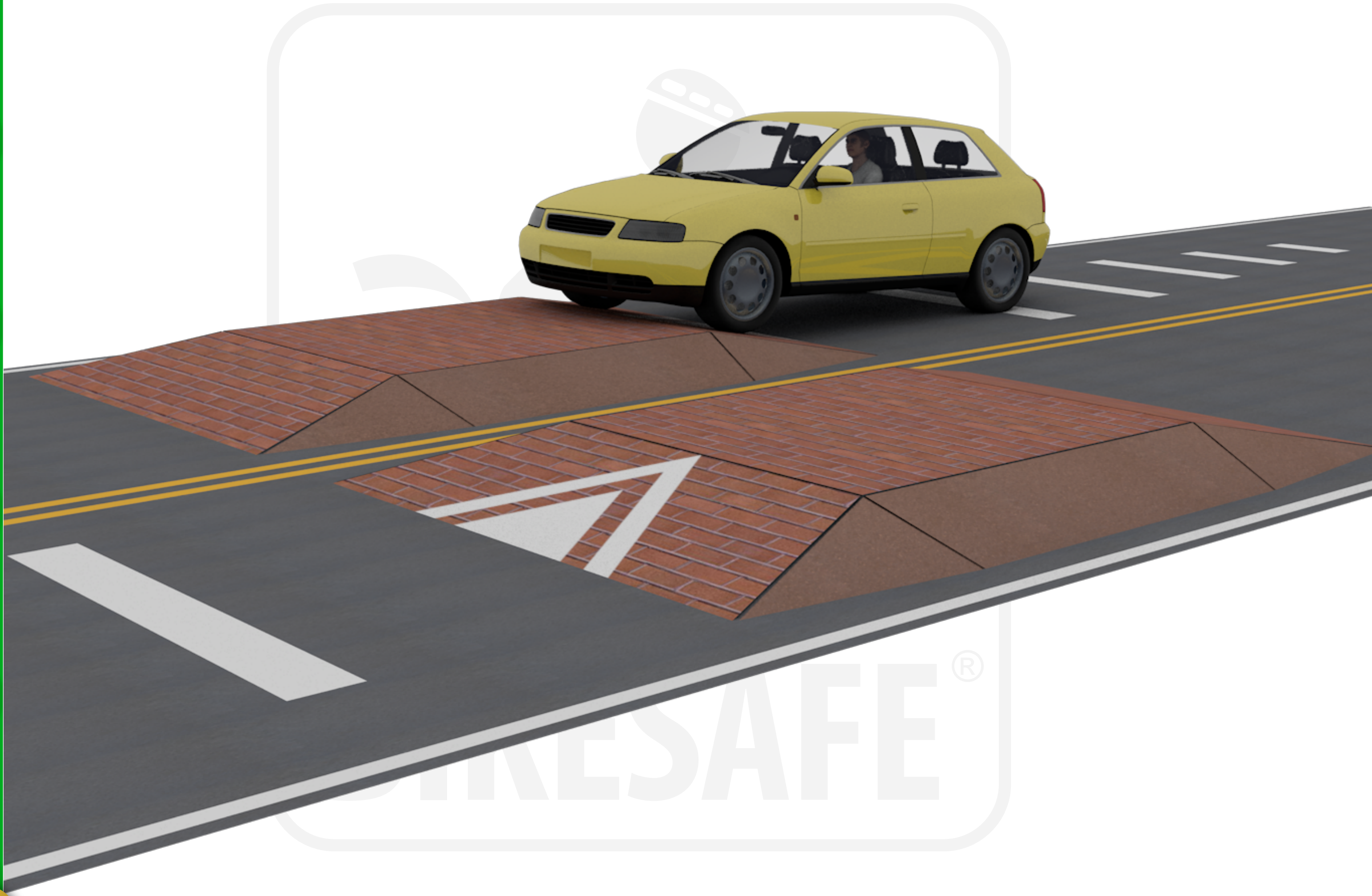

Raised crosswalk

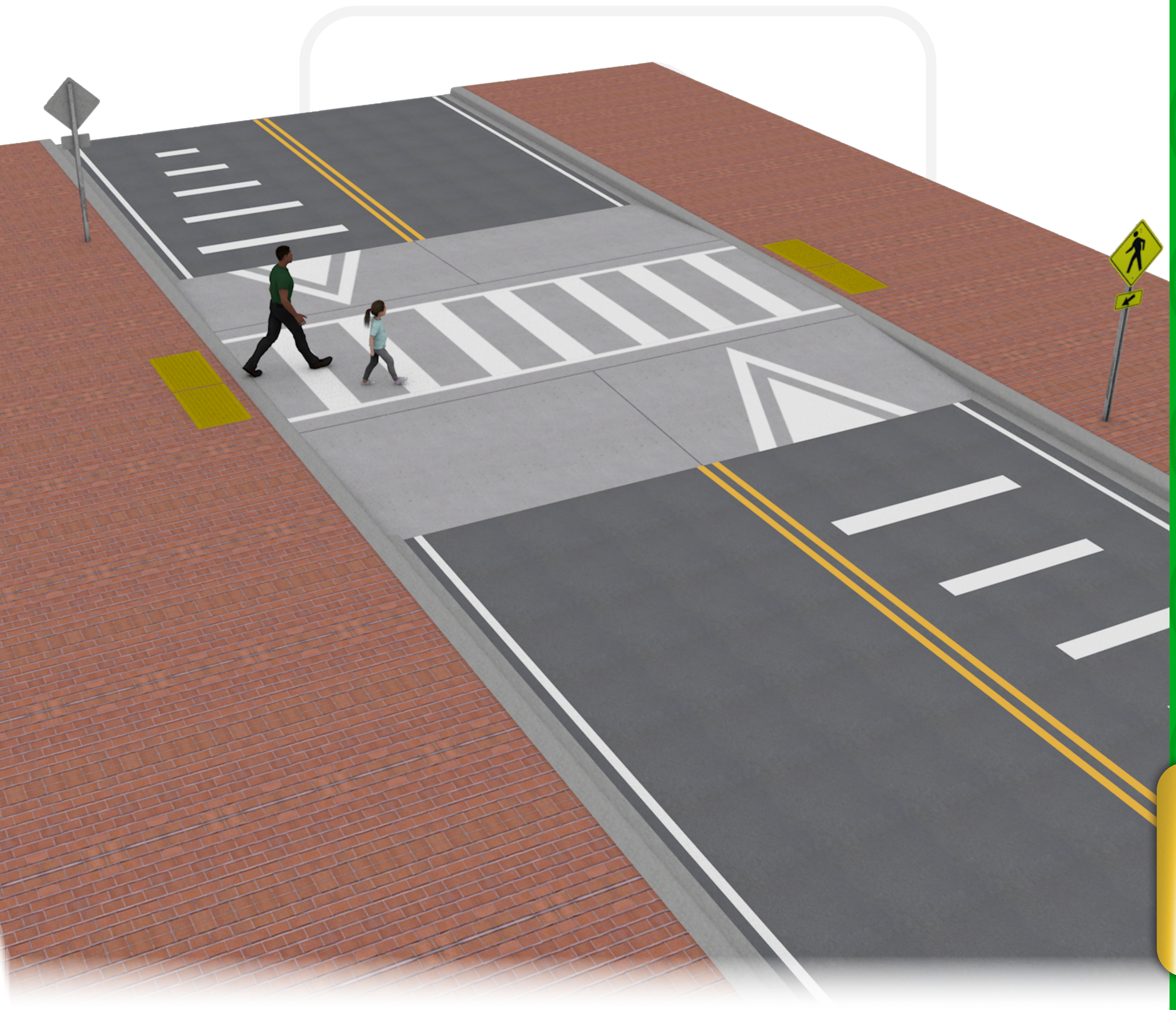

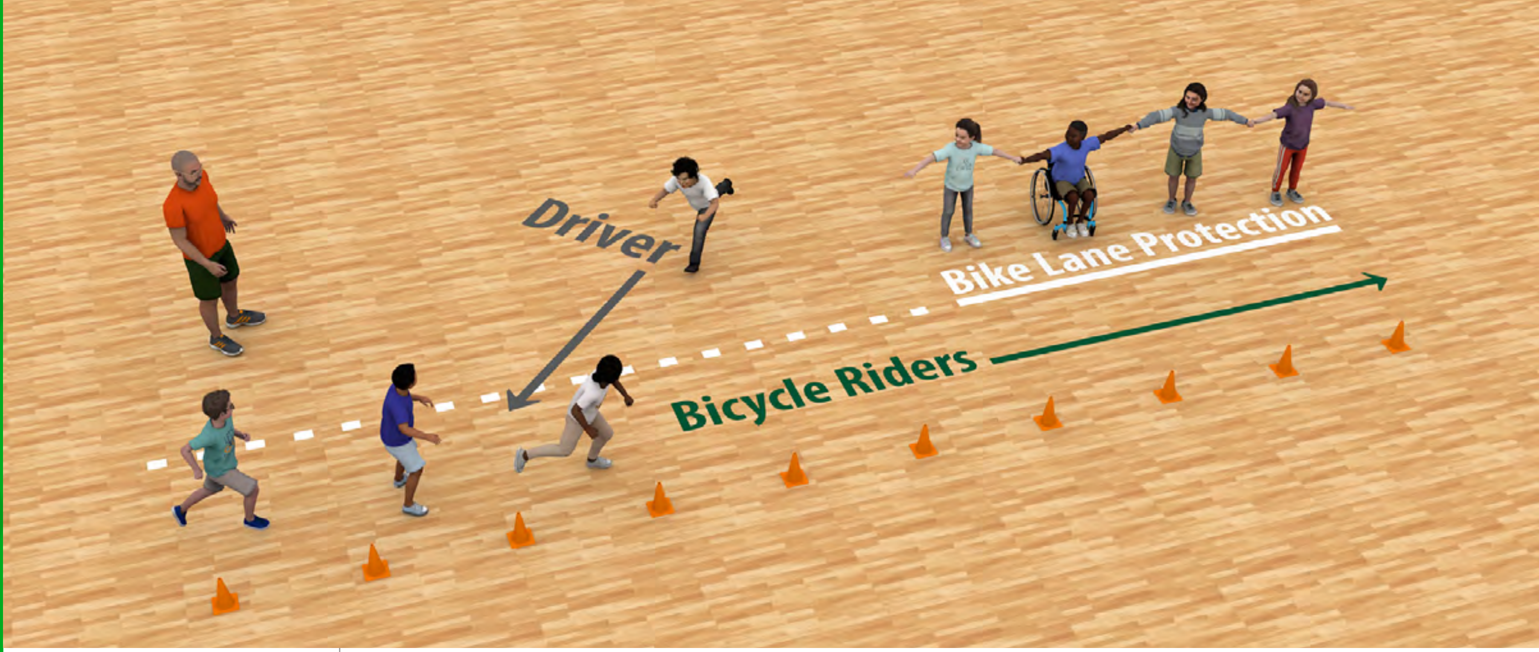

Activity - Sharks and Minnows

(15 minutes)

Protected bike lanes *prevent drivers from injuring bicycle riders. In this activity, students will experience this first-hand.*

To play:

1. Students line up on an "bike lane**"** in your field or court as "bicycle riders." One student is designated as a "driver."

2. Have the riders to run across the court. Instruct the driver to tag as many riders as possible.

3. Tagged students will link together to become a **protected bike lane**. The driver is not allowed to pass this row of students because they are now a protective barrier for the remaining riders.

4. As more students get tagged, the protective "wall" will grow, making it more difficult for the driver to tag bicycle riders.

Optional:

- Instead of running, have students dribble a basketball while walking.
- Add protection with cones - one cone every time a student is tagged.

Module Closure

(5 minutes)

Please remind your students of these key concepts before dismissing class:

- A bike lane is an area made specifically for bicycle riders. When protective barriers are added to a bike lane, we call it a **protected bike lane**. These barriers make it a safer place to ride because riders are separated from drivers.

- **Safe streets** are places where people of any age can walk, roll, and ride bicycles in safety.

- **Traffic calming** measures encourage drivers to slow down for the safety of others, including speed humps and protected bike lanes.

Instructor Notes

Final Thoughts

We hope teaching the BikeSafe Elementary Curriculum was rewarding and easy to implement.

You can help your school community become more active by promoting biking to school, hosting a Bike to School Day event, organizing a "bike bus" (a group of students traveling to school on bike, led by parents and/or teacher volunteers), gathering Safe Routes to School travel tallies, reviewing the bicycle facilities at and around your school, and advocating for safe streets.

Miami-Dade Teachers

Upon completing all modules with your classes, please make sure to submit a Curriculum Completion Form at:
ibikesafe.org/ccf

Bike to School Day

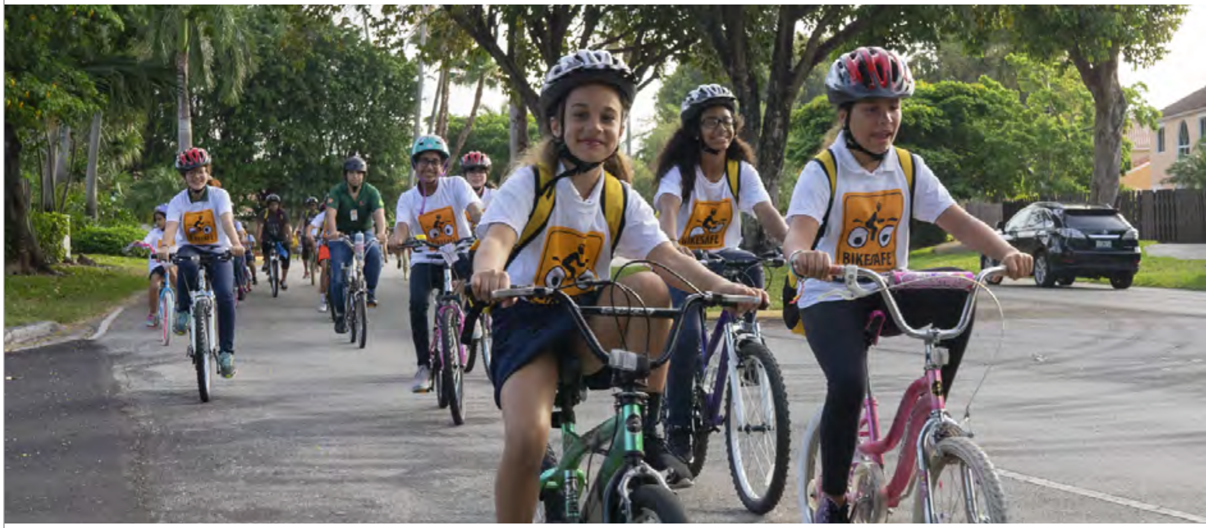

Bike to School Day events are a popular way to encourage youth physical activity, reduce school zone congestion, and feature new protected bike lanes. They can also be used to bring awareness and display demand for protected bike lanes if your area does not have them.

These rides can take on various forms:

1. A grassroots effort where parents and students in the surrounding neighborhood ride to school individually.

2. A grassroots effort where families meet at a specific meeting location and ride as a group to school (a “**bike bus**” or “**bike train**”).

3. An organized ride held by the school, where all families arrive at a predetermined location to ride to the school.

Since not every neighborhood-to-school route is equipped with calm neighborhood streets and protected bike lanes to connect larger roads, school administrators may wish to engage school resource officers and local law enforcement to protect the ride.

If this is of interest to you, BikeSafe offers a complete planning guide tailored for schools seeking to hold such an event.

BikeSafe's Bike to School Day guide is free and is available at:
kidzneurosciencecenter.com/downloads/biketoschoolguide/

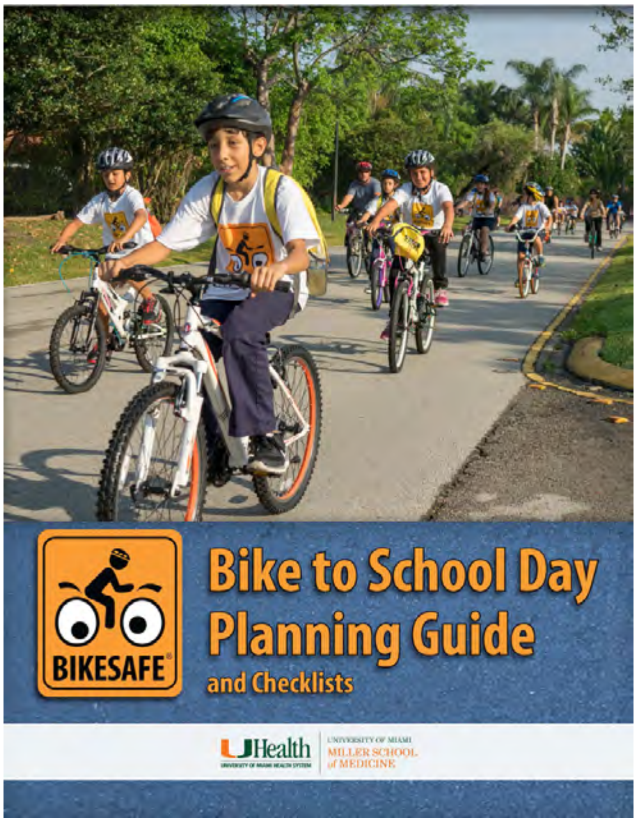

A great Bike to School Day success story is @CoachBalto of Portland, Oregon. He organizes a regular "**bike bus**" to Alameda Elementary School, and is presently inspiring others throughout the US to follow suit.

Mr. Balto has additional resources available at:
linktr.ee/coachbalto

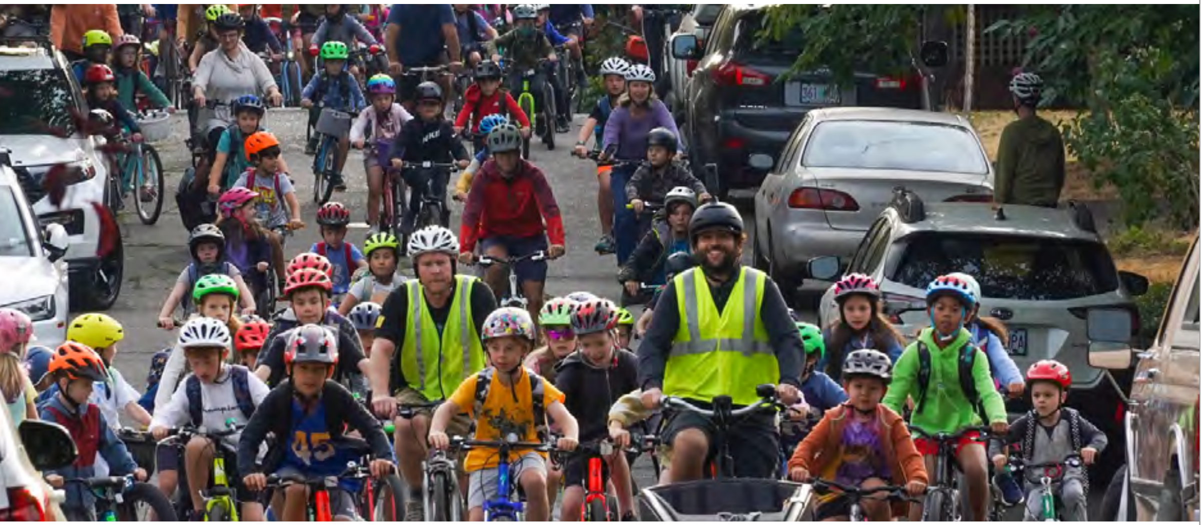

Alameda Elementary School Bike Bus - September 2022. Photo: Jonathan Maus, BikePortland.

Standards

The BikeSafe Program satisfies the following physical education standards:

- **SHAPE America National Standards:**
  shapeamerica.org/standards/pe/

- **Florida State Standards:**
  Grade 4: cpalms.org/PreviewCourse/Preview/898
  Grade 5: cpalms.org/PreviewCourse/Preview/899

Appendix

Access these links, plus additional resources for your school, online:

- **Get Moving on Bike to School Day**
  Action for Healthy Kids
  actionforhealthykids.org/activity/get-moving-on-bike-to-school-day/

- **Making neighborhoods better with protected bike lanes**
  BikeSafe
  kidzneurosciencecenter.com/making-neighborhoods-better-protected-bike-lanes/

- **ChangeLab Solutions**
  Safe Routes to School Policy Workbook for School Districts
  changelabsolutions.org/healthy-neighborhoods

- **Designing for All Ages & Abilities**
  National Association of City Transportation Officials (NACTO)
  nacto.org/publication/urban-bikeway-design-guide/designing-ages-abilities-new/

- **How To Make School Zones Safer with Protected Bike Lanes**
  Safe Kids Worldwide
  safekids.org/blog/how-make-school-zones-safer-protected-bike-lanes-and-have-fun-doing-it

- **In Austin, a Protected Bike Lane Built to Help Kids Get to School**
  Streetsblog USA
  usa.streetsblog.org/2014/01/13/in-austin-a-protected-bike-lane-built-to-help-kids-get-to-school/

- **What is Tactical Urbanism?**
  Tactical Urbanist's Guide
  tacticalurbanismguide.com/about/

- **Planning a Walk or Bike to School Day**
  UNC Highway Safety Research Center
  walkbiketoschool.org/plan/how-to-plan/

- **Promote Walking and Bicycling**
  Vision Zero for Youth
  visionzeroforyouth.org/wp-content/uploads/2019/10/2024_VZY_4-pager.pdf

Relevant Studies:

- Chandler, J., Flynn, J., Bassett, D., Aaron, K., Walsh, J., Manuel, K., Fernandez, R., Epperson, B., & Zavisca, E. (2015). A Community-Based After-School Program to Promote Bicycling Skills and Knowledge: Kids Can Bike! *Journal of Park and Recreation Administration, 33*(4), 90-99. https://doi.org/10.18666/JPRA-2015-V33-I4-6083

- Hamann, C., & Conrad, A. (2019). Inventory of child bicycle education programs reveals need for age, development, and skill-level considerations. *Traffic Injury Prevention, 20*(sup3), 33-38. https://doi.org/10.1080/15389588.2019.1665651

- Hooshmand, J., Hotz, G., Neilson, V., & Chandler, L. (2014). BikeSafe: evaluating a bicycle safety program for middle school aged children. *Accident Analysis and Prevention*, *66*, 182-186. https://doi.org/10.1016/j.aap.2014.01.011

Notes

Notes

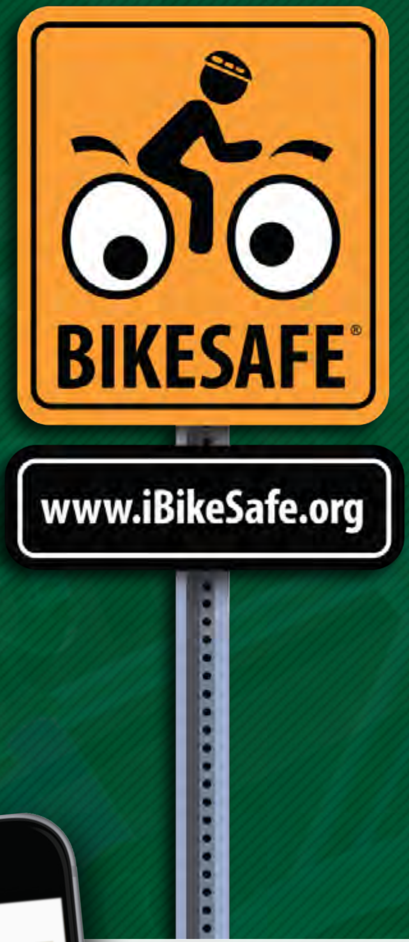

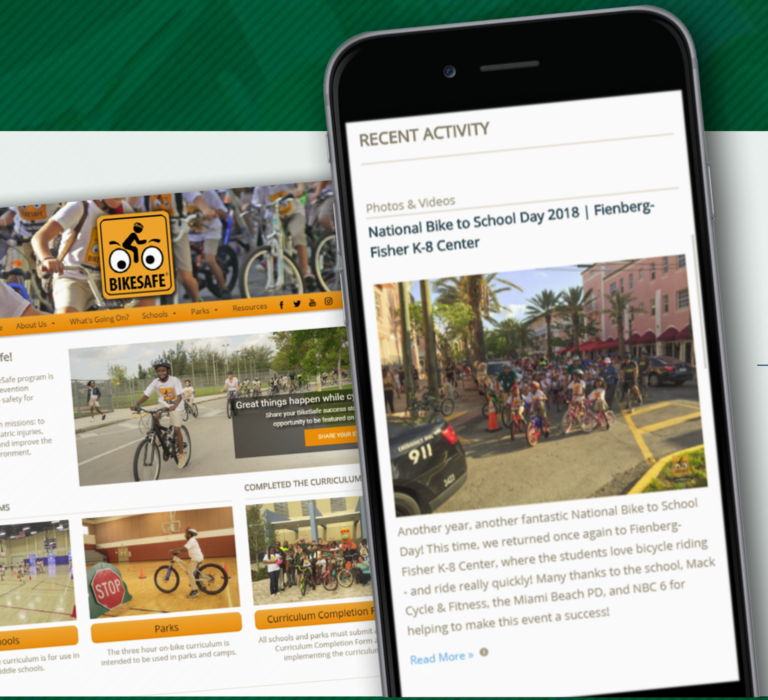

Join the conversation about safer streets:

@iBikeSafe

The BikeSafe Program is brought to you in part by:

FDOT

United States of America Department of Transportation

TPO Miami-Dade Transportation Planning Organization

©2024 University of Miami BikeSafe Program. All rights reserved.
