# Supplementary material for: Evaluation of a Curriculum‐Based Elementary School BikeSafe Program
Source: J Sch Health. 2026 May 5;96:e70162. doi: 10.1111/josh.70162 (PMC13143855; doi:10.1111/josh.70162)
Supplement: Supplementary file 2 — Table S1: School‐level demographic and socioeconomic characteristics of participating schools. School‐level demographic composition of participating schools, including student race/ethnicity, total enrollment, eligibility for free and reduced‐price lunch, and student sex distribution. These data provide contextual information on the socioeconomic and demographic diversity of the school populations included in the study. [file JOSH-96-0-s001.docx]

**Supplemental Table S1.** *School-Level Demographic and Socioeconomic Characteristics of Participating Schools.* School-level demographic composition of participating schools, including student race/ethnicity, total enrollment, eligibility for free and reduced-price lunch, and student sex distribution. These data provide contextual information on the socioeconomic and demographic diversity of the school populations included in the study.

|  | **American Indian/Alaska Native** | **Asian** | **Black** | **Hispanic** | **White** | **Native Hawaiian Pacific Islander** | **Two or more Races** | **Total Students** | **Free and reduced-price lunch eligible** | **% of school population eligible for free and reduced price lunch** | **Male** | **Female** |
| --- | --- | --- | --- | --- | --- | --- | --- | --- | --- | --- | --- | --- |
| **DR. ROBERT B. INGRAM ELEMENTARY SCHOOL** | 0 | 0 | 200 | 73 | 0 | 0 | 0 | 273 | 206 | 75% | 155 | 118 |
| **BROADMOOR ELEMENTARY SCHOOL** | 0 | 0 | 49 | 457 | 2 | 0 | 0 | 508 | 298 | 59% | 266 | 242 |
| **HENRY S. WEST LABORATORY SCHOOL** | 0 | 10 | 31 | 221 | 85 | 0 | 10 | 357 | 68 | 19% | 200 | 157 |
| **CHARLES R. DREW K-8 CENTER** | 0 | 0 | 412 | 42 | 2 | 2 | 0 | 458 | 367 | 80% | 219 | 239 |
| **MIAMI GARDENS ELEMENTARY SCHOOL** | 0 | 0 | 87 | 164 | 10 | 0 | 0 | 261 | 192 | 74% | 138 | 123 |
| **MIAMI LAKES K-8 CENTER** | 0 | 6 | 52 | 1,126 | 35 | 2 | 2 | 1,223 | 589 | 48% | 638 | 585 |
| **SUNNY ISLES BEACH K-8** | 1 | 30 | 21 | 524 | 1,138 | 0 | 22 | 1,736 | 601 | 35% | 882 | 854 |
| **KENDALE ELEMENTARY SCHOOL** | 0 | 9 | 13 | 303 | 17 | 0 | 1 | 343 | 142 | 41% | 176 | 167 |
| **LEEWOOD K-8 CENTER** | 0 | 5 | 34 | 403 | 90 | 0 | 6 | 538 | 158 | 29% | 279 | 259 |

Note: The percentage of students eligible for free and reduced-price lunch varies by school (19%–80%); district-wide eligibility in Miami-Dade County Public Schools is approximately 73%.
